# mi-Mic: a novel multi-layer statistical test for microbiota-disease associations

**DOI:** 10.1186/s13059-024-03256-0

**Published:** 2024-05-01

**Authors:** Oshrit Shtossel, Shani Finkelstein, Yoram Louzoun

**Affiliations:** Department of Mathematics, Bar-Ilan University, Ramat Gan, 52900 Israel

**Keywords:** Cladogram, Nested ANOVA, Image-microbiome, 16S, WGS, Microbiota

## Abstract

mi-Mic, a novel approach for microbiome differential abundance analysis, tackles the key challenges of such statistical tests: a large number of tests, sparsity, varying abundance scales, and taxonomic relationships. mi-Mic first converts microbial counts to a cladogram of means. It then applies a priori tests on the upper levels of the cladogram to detect overall relationships. Finally, it performs a Mann-Whitney test on paths that are consistently significant along the cladogram or on the leaves. mi-Mic has much higher true to false positives ratios than existing tests, as measured by a new real-to-shuffle positive score.

**Supplementary Information:**

The online version contains supplementary material available at 10.1186/s13059-024-03256-0.

## Background

Extensive research has shed light on the intricate interplay between the human host and its resident microbial communities, which play pivotal roles in various physiological processes [1–5]. Current prevailing technologies for microbiome analysis use metagenomic sequencing, where either the DNA (see Acronym table in Additional file 1: Table S1) of a taxonomically informative gene (e.g., 16S rRNA) or all the genomic DNA in the microbial genome is sequenced (e.g., whole genome sequencing (WGS)). The raw sequencing reads are clustered into operational taxonomic units (OTUs), denoised into amplicon sequence variants (ASVs), or mapped to a microbial reference database (taxa) using existing bioinformatics pipelines, such as DADA2 [6] (for 16S), and MetaPhlAn [7, 8] (for WGS). For the sake of clarity, we use the term taxon to represent any taxonomic unit (OTU/ASV/taxon) from a bioinformatics pipeline [6, 9, 10]. Differential abundance analysis can then be carried out based on the processed taxa abundance and metadata table to associate microbes with different conditions (labels).

Differential abundance analysis aims to find the differences in the abundance of each taxon between multiple classes of subjects or samples, assigning a significance value to each comparison, or associating the taxon with a continuous variable. We use the following notation in the current analysis: $x_{i}^{j}$ is the $j_{th}$ taxon of the $i_{th}$ sample, and $y_i$ denotes the sample’s label that can be binary, categorical or continuous (e.g., presence or absence of a disease, the host BMI or the source of the sample). The goal of abundance analysis is to explore whether the label $y_i$ correlates with the abundance of a particular taxon or group of taxa under different labels. We focus in the description on binary labels, but the method presented here can be similarly applied to categorical variables.

The main current differential analysis methods include LEfSe [11], ANCOM [12], ANCOM-BC2 [13], DeSeq2 [14], ALDEx2 [15, 16], and LINDA [17]. Other less used methods were also proposed [18–21]. LEfSe employs the Kruskal-Wallis sum-rank test [22] to identify features with significant differential abundance, followed by Wilcoxon rank-sum tests [23, 24] for biological consistency. It then uses linear discriminant analysis (LDA) [25] for effect size estimation. ANCOM operates on log-normalized data with a pseudo-index to handle zero values. It then calculates pairwise log ratios, conducts significance tests using Kendall’s W statistic, and applies a Bonferroni correction for multiple comparisons. ANCOM-BC2 extends ANCOM by incorporating additional blocking and covariate terms into the model through multivariate regression analysis. DeSeq2 models variance-mean dependence in count data using the negative binomial distribution. ALDEx2 employs Monte Carlo sampling and Bayesian modeling to estimate technical variability and sampling uncertainty in compositional microbiome data. LINDA performs a linear regression with CLR (centered log ratio)-transformed abundance data, corrects for compositional bias, calculates *p*-values based on bias-adjusted coefficients, and controls the false discovery using the Benjamini-Hochberg procedure.

Among the less common methods are those that incorporate phylogenetic information into their statistical frameworks. For example, structSSI [26] adopts a hierarchical false discovery rate (FDR) control strategy, organizing hypotheses along a phylogenetic tree to enhance the detection of significant signals. Specifically, children’s hypotheses are considered for rejection if and only if their parents are rejected. PhAAT (Phylogeny-aware Abundance Testing), https://github.com/mruehlemann/phaat, constructs a Branch-Abundance matrix by multiplying the sequence abundance table with the binary Sequence-to-Branch matrix, enabling representation of branch abundance. PhAAT then conducts filtering steps to reduce multiple testing burdens, applies statistical tests for differential abundance, refines signals by clustering sub-branches with the same effect direction, and annotates final signals based on taxonomic annotations weighted by abundance. ada-ANCOM [27] extends the Dirichlet-tree multinomial (DTM) to zero-inflated DTM, addressing data sparsity (like ANCOM-2) and incorporating phylogeny. It introduces a Bayesian formulation with posterior mean transformation to convert raw counts into non-zero relative abundances, facilitating adaptive analysis of the composition of microbiomes for differential abundance testing (Table 1).

**Table 1:** Comparison between existing differential analysis methods considering the “Normalization” — normalization applied during the method, “Normality assumption” — whether the method assumes the microbiome input is normally distributed, “Inner relations” — how the method treats the inner relations between the taxa, and “Multiple measurements” — how the method treats the problem of multiple measurements

Model	Normalization	Normality assumption	Inner relations	Multiple measurements
LEfSe	Relative abundances	X	X	X
		Kruskal-Wallis		
ANCOM	Log normalization	X	X	V
		Kendall’s W analysis		Bonferroni correction on initial results
ANCOM-BC2	Additive Log-Ratio (ALR) transformation	X	X	V
		Wilcoxon rank-sum	But fixes for covariates	Bonferroni correction on initial results
DeSeq2	Negative Binomial distribution	V	X	V
		Wald test		Bonferroni correction on initial results
ALDEx2	CLR transformation	X	X	V
		Wilcoxon rank-sum	But fixes for covariates	Benjamini Hochberg correction on initial results
LINDA	CLR transformation	V	V	V
		OLS regression	OLS Regression	Benjamini Hochberg correction on initial results
structSSI	None	V	V	V
		Linear model	HFDR correction	HFDR correction
PhAAT	None	V	V	V
		Linear model	Filtering according to similarity of child branches	1. Filtering out branches with low abundance
				2. Filtering out branches that differ too little from their child branches (Bray Curtis)
ada-ANCOM	Log normalization	1/2	V	V
		*t*-test or Wilcoxon rank-test	Cladogram	Through the cladogram
miMic	Supports both log normalization and relative normalization	X	V	V
		Mann-Whitney	Cladogram	1. A priori nested ANOVA
				2. Through the cladogram
				3. Sister correction
				4. First layer correction
				5. Leaves correction

Differential analysis methods face three primary challenges: (1) dealing with non-normally distributed microbial data (specifically, most taxa in most samples with 0 values, and a heavy tail distribution for the non-zero taxa), (2) mitigating high false discovery rates, and (3) accounting for inherent relationships between microbial taxa, which introduce measurement dependencies.

Microbial frequencies span a broad distribution encompassing extremely rare and highly prevalent taxa [28, 29]. Moreover, most microbes are absent from most samples. Thus, even after applying log normalization to address the wide distribution, the data often exhibits a non-normal, bimodal distribution [30–33] (Additional file 1: Fig. S1). Consequently, the application of parametric tests on processed microbial taxa may be inaccurate. Some existing methods address this challenge by resorting to non-parametric tests, such as the Kruskal-Wallis test [22] in LEfSe [11] Kendall’s test [34] in ANCOM [12], or Wilcoxon rank-sum test in ANCOM-BC2 [13] and ALDEx2 [15, 16] while others, like DeSeq2 [14] and LINDA [17], overlook the normality concern (Table 1).

The high dimension of microbial data increases the risk of false discoveries [17, 35, 36]. Many existing methods attempt to mitigate this issue through various multiple-measurements corrections, including the Bonferroni [37] correction in ANCOM and DeSeq2 or the Benjamini-Hochberg correction [37] in ALDEx2 and LINDA. However, these corrections often prove overly stringent, resulting in a reduction of true positive signals alongside false positive rate reductions (Table 1).

Another assumption common to most existing methods, such as LEfSe, ANCOM, and DeSeq2 is the independence of tests over all taxa. However, often, similar taxa exhibit similar behavior, as extensively discussed [18, 38–40] and further shown below. These intrinsic taxonomic relationships challenge the independence assumption. LINDA addresses these correlations through initial regression on taxa (Table 1).

However, the intrinsic taxonomic relationships can be used to mitigate the problem of too many independent tests, since the tests are not independent.

To introduce the relation between taxa in relative abundance analysis and simultaneously solve the multiple measurement corrections, we here introduce mi-Mic (Mann-Whitney iMage Microbiome, further referred to as miMic) — a novel framework to apply differential abundance analysis to the non-normally distributed microbial data (Table 1).

Simply stated, on the one hand, applying a Bonferonni multiple measurement correction on all taxa is too stringent. On the other hand, applying a test on all taxa with no correction can produce many false positive associations (basically, the *p*-value multiplied by the number of taxa, which is often more than the number of biologically significant associations). However, formally, not all taxa are independent measurements, since the frequencies of similar taxa are often correlated. miMic uses these relations and a simplifying assumption that if a taxon is associated with a label, this effect is strong enough to affect the average of the taxa at coarser levels to reduce the number of required corrections. In practice, miMic performs the correction at coarse levels that have few taxa, and then only performs tests along significant paths in the cladogram.

**Fig. 1:**
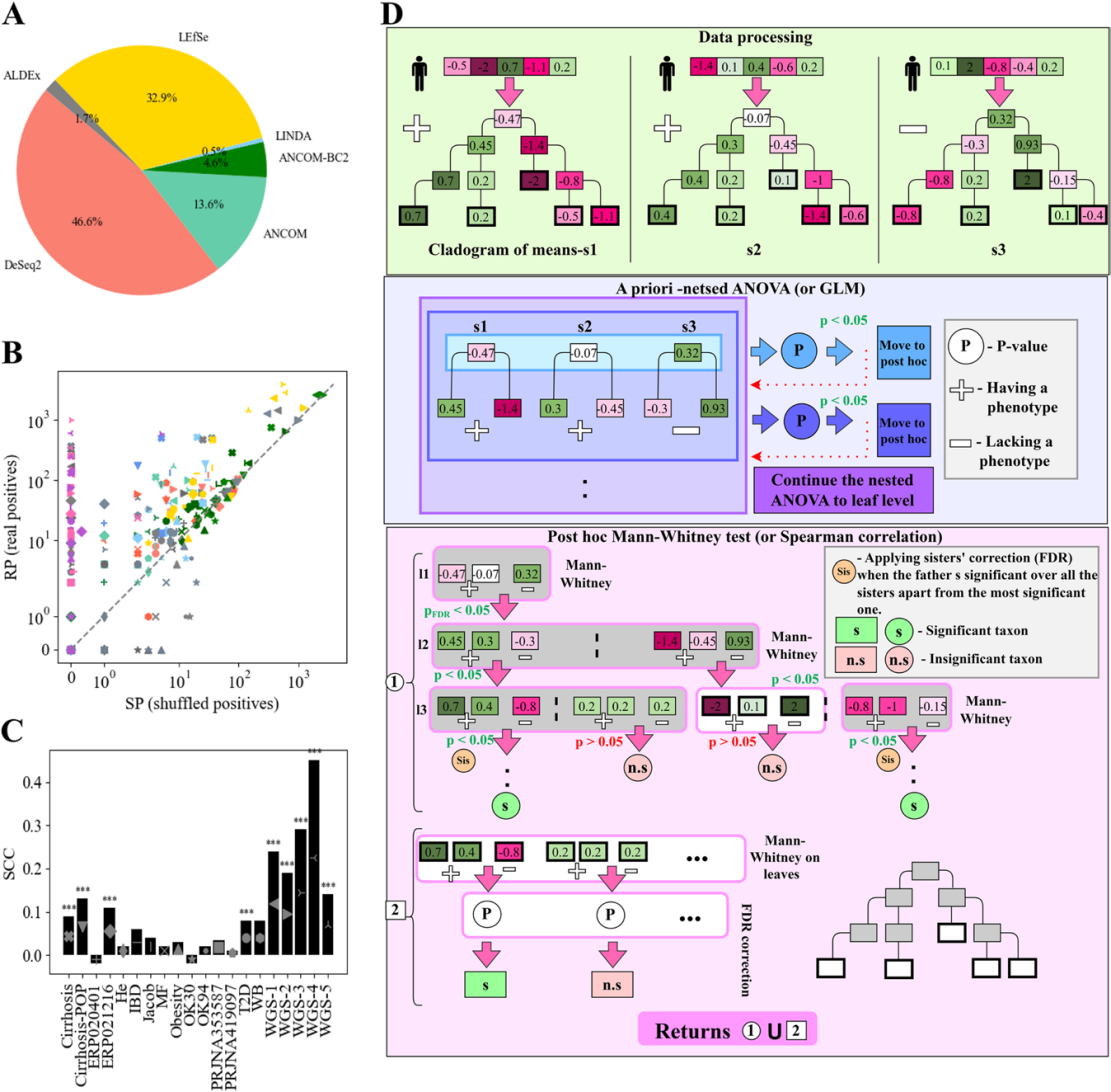
Challenges in difference analysis of microbiome **A** Pie diagram illustrating the usage of differential analysis methods within the field during 2023. The colors represent different methods: LefSe (yellow), DeSeq2 (orange), ANCOM (sea-green), ANCOM-BC2 (green), ALDEx2 (gray), and LINDA (light blue). **B** Scatter plot comparing the performance (SP vs. RP) of popular methods across more than 20 different microbial datasets. Each shape represents a distinct cohort with colors matching those indicated in the pie chart (**A**). Dark colors indicate methods with FDR corrections, while light colors represent methods without any corrections. The dashed gray line represents the $y = x$ line, where the SP rate is similar to the RP rate. Pink and purple colors in the upper left corner represent miMic and miMic-relative, respectively. In many cases (excluding miMic), the RP and SP rates are similar. **C** Inner sisters’-labels SCCs over the different datasets. The stars represent the significance of the correlations, such that *-$p<0.05$, **-$p<0.01$, ***-$p<0.001$. **D** Schematic explanation of the miMic approach. miMic is a three-step method: (1) Data processing (in light green). Microbiome data preprocessing involves using the MIPMLP method (see the “Methods” section) and converting it into a cladogram of means using the iMic algorithm (see Methods). (2) A priori nested ANOVA (in light blue). An a priori nested ANOVA (nested GLM for continuous labels) is applied to microbiome cladograms and labels to assess the global relationship between labels and the entire microbiome. If no significant relationship is found at any taxonomy level, the label is defined “not microbially explainable,” and no further analysis is performed. (3) Post hoc Mann-Whitney test. A post hoc Mann-Whitney test, leveraging phylogenetic information, is conducted for the difference analysis (1). Starting at the first taxonomy level, a Mann-Whitney (or Spearman correlation) test is applied to each taxon’s values, with a predetermined significance level (currently $p < 0.05$). Multiple measurement correction is applied only for the number of taxa at this level. If significant taxa are identified, the analysis iteratively proceeds along the cladogram towards the leaf nodes. To address inner sister relations, FDR control is used when multiple significant sisters are present. An additional Mann-Whitney test (utilizing Spearman correlation for continuous labels) is specifically conducted over the leaves, incorporating FDR correction for multiple measurements (2). miMic consolidates the significant taxa identified in steps 1 and 2. The schematic cladogram in the bottom right corner illustrates the inner taxa of the cladogram utilized in step 1 of the post hoc test (gray), while its white leaves are employed in both steps. “+/−” denotes samples with/without the phenotype, respectively. A white circle marked “p” represents a *p*-value. An orange circle labeled “Sis” signifies FDR correction applied over the sisters, as explained in the manuscript. A green square or circle labeled “s” indicates a significant taxon, while a red square or circle marked “n.s” indicates an insignificant taxon

More specifically, miMic first applies normalization and translation of ASV to log-normalized taxa frequencies using the MIPMLP pipeline [41]. These taxa are combined to a cladogram of means[42] (see detailed explanation in the “Methods” and Fig. 1D Data processing step). Then an a priori phylogeny aware test (nested ANOVA or parallel nested Generalized Linear Model (GLM) for continuous labels) is applied to the cladograms to test whether there is any microbiota-label association in the cohort (Fig. 1D a priori nested ANOVA test). If the a priori test is significant, a phylogeny-aware Mann-Whitney (Spearman correlations for continuous labels) test (non-parametric since the distributions of the observations are often non-normal) along trajectories of the cladogram is applied to identify the specific significant taxa (Fig. 1D post hoc miMic test 1). Subsequently, an additional Mann-Whitney test (Spearman correlation for continuous labels) is conducted solely over the leaves, with FDR correction for multiple measurements (Fig. 1D post hoc miMic test 2). miMic returns the significant taxa found in 1 and 2. miMic combines a parametric test on the coarser levels of the mean cladogram, since those converge to a normal distribution, with a non-parametric test on the leaves (the log observed frequencies). However, miMic may miss rare species that are strongly associated with a label but have practically no effect on coarser taxa. To address that miMic also includes significant leaves after a multiple measurement correction.

A challenge in the comparison of differential analysis methods is the absence of a robust ground truth (GT). In real-world datasets, there is typically no GT for association detected. A conventional approach involves employing permutations, wherein labels are shuffled multiple times, and the resulting presumed false-positive (FP — the number of significant associations within the shuffled data) is compared against a predetermined expected error rate[17]. However, permutation-based evaluations tend to prioritize error reduction, sometimes at the expense of actual discoveries, since the true associations are unknown, and as such, one cannot penalize missed associations (false negatives (FN)).

To address this limitation, we introduce the RSP score (real positives vs. shuffled positives), which represents the ratio between real positives (RP) and shuffled positives (SP) as a function of the confidence parameter, $\beta$, offering a more comprehensive perspective. This novel scoring metric optimizes both the identification of real positives and the control of shuffled positives.

## Results

### Challenges in difference analysis of microbiome

As mentioned, a plethora of approaches were proposed for difference analysis in microbiome [11–15, 17], each striving to identify meaningful taxonomic variations. Here, we focus on the six most widely-used and well-cited methods (defined by the number of citations within 2023 in over 30,000 published manuscripts), namely LEfSe, ANCOM, ANCOM-BC2, DeSeq2, ALDEx2, and the innovative LINDA (Fig. 1A).

Given the absence of a definitive GT, we cannot use true positives (TP) and false positives (FP) to compare methods. Instead, we compared the “real positives - RP”, the number of significant taxa in the observed data with the real labels, to the “shuffled positives - SP” — the number of significant taxa when the test is applied on the same microbial data, but shuffled labels. We compared 20 diverse datasets comprising both 16S (12 datasets) and WGS data (8 datasets) (Fig. 1B and Additional file 1: Table S2). Most, but not all popular approaches exhibited higher numbers of RP than SP (Fig. 1B). However, in most methods, there was a very high number of SP, and often, a similar order of RP and SP (diagonal in Fig. 1B). Applying multiple measurement corrections does not improve the RP to SP ratio, since the reduction in SP often comes at the expense of a drastic decrease in RP, or in some cases, has a minimal impact on SP (Fig. 1B, darker colors).

The second potential error is the taxa independence assumption. To address this concern, we define sisters as taxa having the same “mother” in the cladogram (e.g., *Bifidobacterium (s)* and *adolescentis (s)* are sisters that share the same mother *Bifidobacterium (g)*). Approximately half of the datasets demonstrated significant sister-label relations, as defined by the Spearman correlation coefficient (SCC) between the normalized frequency, at a significance level of $p < 0.05$ (Fig. 1C and Methods for details).

### miMic: a phylogeny-informed approach to address microbiome difference analysis challenges

To address the challenges above, we propose a comprehensive 3-step approach named miMic. miMic incorporates phylogeny information to enhance the accuracy and reliability of microbiome difference abundance analysis. We outline the key steps of miMic and its potential benefits, before showing the validity of the assumptions underlying and its high accuracy on analytical models, simulations, and real-world data.

As the first step, miMic preprocesses the microbiome frequencies using MIPMLP [41] (see Methods) and translates them into a cladogram of means using the iMic algorithm [42] (Fig. 1D data processing). This cladogram captures the taxonomic relationships within the microbiome data, providing valuable insights into the underlying structure.

The second step is an a priori nested ANOVA test to the microbiome cladograms and the labels to test for a relation between the label and the microbiota (Fig. 1D a priori nested ANOVA). If no significant relation is identified at any taxonomy level, the label is deemed “not microbially explainable,” and no further difference analysis is performed. If the label is continuous, the ANOVA is replaced by the appropriate GLM.

As a final step, a post hoc Mann-Whitney test is applied that leverages phylogeny information to conduct the difference analysis. Starting with the first taxonomy level, a Mann-Whitney (or Spearman correlation) test is applied to the values of each taxon, with a predetermined significance (currently $p<0.05$). Multiple measurement corrections are employed, but only for the number of taxa at this level. If significant taxa are detected, the test proceeds iteratively along the cladogram towards the leaves (Fig. 1D post hoc Mann-Whitney test). Notably, no multiple measurement correction is applied beyond the first level due to the stringent demand for significance along the entire cladogram trajectory. At each such level, the number of candidate taxa is low. If no significant taxa are found, we repeat the analysis starting with a finer taxonomy level (until the class level). To account for inner sister relations, we employ FDR when multiple significant sisters are present. When one sister and its significant sister share the same “mother” in the cladogram, FDR is applied to the *p*-values of all sisters, except the most significant one. To handle rare species, miMic also defines significant leaves following multiple measurement corrections as significant, even if they have no significant ancestor. Since, previously we have shown that those contribute practically no shuffled positives.

By integrating phylogeny information, miMic offers several advantages over existing approaches. The requirement for significance along the cladogram trajectory effectively reduces the false discoveries rate. Additionally, miMic’s consideration of inner sister relations ensures more reliable detection of real positive associations without drastically decreasing their number. Since sister taxa are correlated, there is a high probability that if a taxon is associated with a label, so will be the average with its sisters (mother taxon), as shall be proven.

### Validation of miMics’ assumptions on analytical models, simulations, and real-world data

#### Analytical models

To understand the logic behind miMic, assume three sister taxa with a common mother with their average in each sample. Further, assume that for each class, the distribution of each sister is normal and that with no loss of generality, the average of all sisters in the first class is 0, and the variance is 1. Each daughter taxa is assigned with a value $x_{ij}$ and the mother taxon is assigned: $m_j=\sum _i x_{ij}/3$. Since we focus on the distribution, we denote for a generic taxon in a generic sample $m=m_j,z_i=x_{ij}$.

One can study three simplified regimes.

- **(0,0,0)** — where there is no difference between the classes, and the average of all sisters in the second class is also 0. $z1,z2,z3\sim \mathcal {N}(0,\,1)$. In such a case, neither the sisters nor the mother should be significant.
- **(0,0,**$\varvec{\mu }$**)** — The last sister has an average of $\mu$ in the second class and is thus associated with the class. $z1\sim \mathcal {N}(\mu ,\,1)$ and $z2,z3\sim \mathcal {N}(0,\,1)$. We would expect both the mother and the last sister to be significant, but none of the others.
- **(0,**$\varvec{\mu }$**,**$\varvec{\alpha \cdot \mu }$**)** — The two last sisters have a difference between the first and second class, with varying strengths. $z3 \sim \mathcal {N}(0,\,1)$, $z1 \sim \mathcal {N}(\mu ,\,1)$, and $z2\sim \mathcal {N}(\alpha \mu ,\,1)$. In this case, one would expect if $\alpha >0$, both cases would be easily detected, but if $\alpha <0$, we may miss them, since their mother may lose the correlation with the label.

**Fig. 2:**
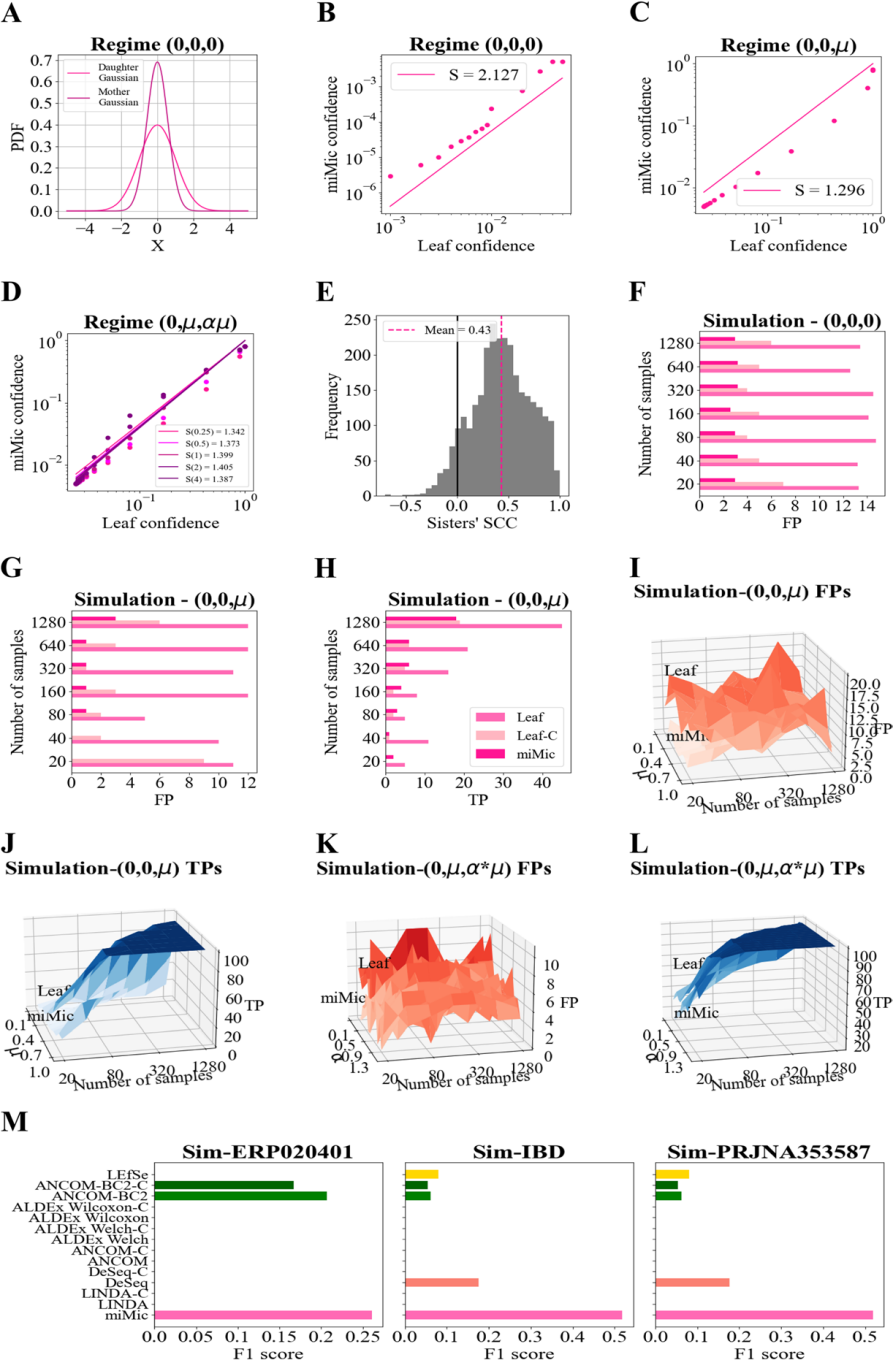
Validation of miMics’ assumptions on analytical models and simulations. **A** Daughter’s distribution (pink) vs. mother’s distribution (dark pink) in the regime of (0,0,0). The mother’s distribution is noticeably narrower, with approximately half that of the daughter’s distribution. **B**–**D** Comparison of the leaf confidence with miMic’s confidence based on analytical integral calculations over 3 different regimes: regime (0,0,0) (**B**), regime (0,0,$\mu$) (**C**), regime (0,$\mu$,$\alpha *\mu$) (**D**). The lines represent estimated slopes, denoted as S. In D, different colors represent varying levels of connection between the sisters, controlled by $\alpha$ values (0.25, 0.5, 1, 2, 4). **E** Histogram illustrating the distribution of inner sisters-label SCCs across different cohorts. The black line represents the zero line, and the dashed pink line represents the average of the distribution, indicating a right-skewed distribution, with most sisters showing a consistent positive correlation with the label. **F**–**G** A comparison between the number of samples and the number of FP for miMic and Mann-Whitney leaf simulations based on the regime of (0, 0, 0) (**F**) and the regime of (0, 0, $\mu$), where $\mu$ was set to 1. The lightest pink color represents the Leaf-C model, the middle pink represents the Leaf model, and the darkest pink represents the miMic model. **H** Comparison between the number of samples and the number of TP for miMic and Leaf Mann-Whitney simulations based on the regime of (0, 0, $\mu$). The color coding is similar to the color coding of **F**–**G**. **I**–**J** Comparison between the FP (**I**) and TP (**J**) of the miMic model and the Leaf model in the simulation of the regime (0, 0, $\mu$) over different numbers of samples and different values of $\mu$.The Leaf model shows a higher number of FP compared to miMic, while miMic’s TP taxa are similar to Leaf’s TP taxa. **K**–**L** Comparison between the FP (**K**) and TP taxa (**L**) of the miMic model and the Leaf model in the simulation of the regime (0, $\mu$, $\alpha *\mu$) over different numbers of samples and different values of $\alpha$. The Leaf model exhibits a higher number of FP compared to miMic, while miMic’s TP are similar to the Leaf’s TP. **M** The F1 scores of different differential abundance methods are depicted across three distinct setups of microbiome-oriented simulations. miMic is again pink and is much higher than all current methods, many of which have an F1 of 0

In this simplified model, one can analytically show (see Appendix and Fig. 2A) that if the sisters are not associated with the class $\sim \mathcal {N}(0,1)$, then their mother distribution is ($\sim \mathcal {N}(0,\frac{1}{\sqrt{(}3)}))$. As such, the probability that it will be observed as significant is even lower than the one of the daughter (formally, this is equivalent to having $6S-2$ degrees of freedom (DOF) and doing a test with $2S-2$ DOF, where *S* is the number of samples). Moreover, numerical results show that requiring a *p* level significance on both the mother and the daughter is approximately equivalent to a $p^2$ requirement on the daughter. As such, the fraction of SP cases is much lower than *p* (see Fig. 2B).

In contrast, when the daughter is associated with the class, then with a very high probability the mother is also associated with the class (except for the cases, where the association of the daughter is marginal, and then the addition of the $\sim \mathcal {N}(0,2)$ from its sisters masks the signal). Thus, performing both tests is almost equivalent to performing only the daughter test. As such the fraction of RP cases is practically not affected if $\alpha > 0$ (Fig. 2C, D).

This still leaves the problem of the other sisters. If one sister is associated with the label, so will their mother with a high probability. As such, the fraction of SP for those may be high. To prevent this case, we apply a multiple measurement correction in this specific case (Fig. 2D).

Finally, the case where miMic may fail is when one sister is positively associated with the class, and the other is negatively associated with the class. However, the fraction of such cases is very small (Fig. 2E). Moreover, to handle such cases, miMic also includes significant leaves following a multiple measurement correction.

#### Simulations

##### Generic hirerachial simulations

We further validated our analytical analysis using simulations designed to mimic the hierarchical structure of our analytical model. These simulations were the counterpart of the analytical models above. In each scenario, we assessed the performance of miMic against two leaf-level Mann-Whitney tests: one with corrections for multiple measurements (referred to as “Leaf-C” and displayed in the lightest pink) and the other without corrections (referred to as “Leaf” and presented in the middle pink). Since those are simulations, we know the GT. Thus, the evaluation criteria encompassed the identification of both FP and TP, alongside the computation of theoretical probabilities of significance.

The simulations were executed across a range of sample sizes (*N* varying from 20 to 1280) and $\mu =0.1-1$. Much like our analytical analysis, the simulation results consistently underscore miMic’s distinct advantages. Notably, miMic consistently demonstrated a lower number of FP while maintaining a comparable TP rate across all scenarios (note that those are simulations, and we know the GT). miMic consistently outperformed the two Mann-Whitney leaf tests (as illustrated in Fig. 2F, G ($\mu$ =1), I, and K). As expected from the analytical models, miMic’s ability to control the false positive rate did not compromise its capacity for true positive detection when compared to the Mann-Whitney leaf tests (Leaf and Leaf-C) under the same simulated labels and hyperparameters (as depicted in Fig. 2H ($\mu$ =1), J, and L).

**Realistic microbiome-based simulations**

To further assess miMic against existing methods, we conducted simulations using three diverse real microbial datasets with varying sample sizes (PRJNA353587, *n *= 83; IBD, *n *= 257; ERP020401, *n *= 684), we aimed to comprehensively capture the characteristics of genuine microbiome data. We randomly selected 10 taxa along with all their ASVs, elevating their abundances by 20% in samples randomly labeled as positive. A parallel process was executed for another set of 10 taxa linked to samples randomly labeled as negative. Sample labeling was independent for each sample and with equal probability for positive and negative. We computed the F1 score for each model (Fig. 2M). Again miMic has a higher F1 score than all current methods.

#### Real-world cases

**Fig. 3:**
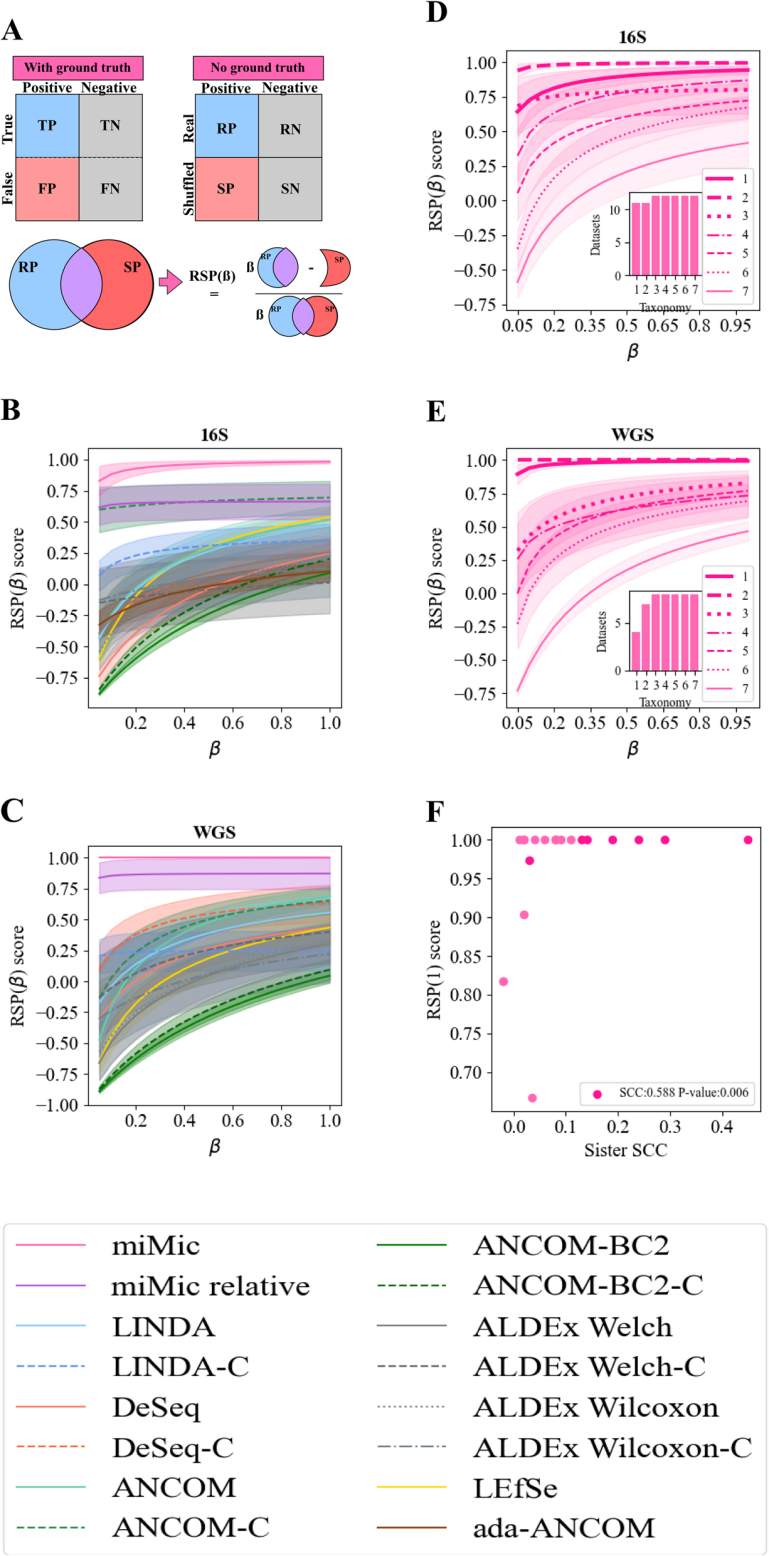
Validation of miMic vs. SOTA models on real-world datasets. **A** RSP($\beta$) schematic explanation. The tasks in the differential analysis field can be divided into 2 main types: (1) tasks with a predefined GT, where False and True labels are clearly defined (upper left scheme), and (2) tasks without a predefined GT, as is common in most real-world datasets (upper right scheme). In the second scenario, “True” and “False” are replaced with “Real” (calculated on real labels) and “Shuffled” (calculated on shuffled labels). The RSP($\beta$) score is defined by $(\beta \cdot RP -SP)/ (\beta \cdot RP+SP))$, where $\beta$ representing the user-defined weighting of Real Positives (RP) vs. Shuffled Positives (SP). **B**–**C** Comparative analysis of different differential analysis methods as a function of RSP($\beta$) over 16S cohorts (**B**) and WGS cohorts (**C**). Each color represents a specific model: orange for DeSeq2 (light without FDR correction and dark with FDR correction, denoted as DeSeq2-C), yellow for LefSe, green for ANCOM (light without FDR correction and dark with FDR correction, referred to as ANCOM-C), blue for LINDA (light without FDR correction and dark with FDR correction, denoted as LINDA-C), brown for ada-ANCOM, and pink for miMic (pink for log Sub-PCA MIPMLP preprocessing and purple for relative mean MIPMLP preprocessing). Each line illustrates the average RSP($\beta$) score across all cohorts (12 16S in **B** and 8 WGS in **C**). The light shadows surrounding each line represent standard errors calculated over 10 simulations of the shuffled models across all cohorts (12 16S in (**B**) and 8 WGS in (**C**)). **D**–**E** Comparison of different starting taxonomy levels of the miMic test and their corresponding RSP($\beta$) scores over 16S cohorts (**D**) and WGS cohorts (**E**). Each taxonomy level is indicated by a different line style (1 for kingdom, 2 for phylum, 3 for class, 4 for order, 5 for family, 6 for genus, and 7 for species). Typically, the best RSP scores are achieved in the first two taxonomy levels. The inner bar plot presents the number of cohorts in which miMic is deemed significant when commencing the Mann-Whitney test at each taxonomy level. **F** Scatter plot of the sister’s-labels SCC vs. the RSP(1) score. A significant positive correlation of 0.588 is observed between the SCCs of sister labels and the model’s performance

To show that miMic is indeed much more accurate than the current state-of-the-art (SOTA) methods, we compared it with the most popular SOTA - LEfSe, DeSeq2, ANCOM, ANCOM-BC2, ALDEx2, and LINDA over 20 different diverse datasets comprising both 16S (12 datasets, see Additional file 1: Table S2) and WGS data (8 datasets, see Additional file 1: Table S2). As mentioned, an inherent challenge when assessing differential analysis over real-world cases is the absence of GT information (illustrated in Fig. 3A). Therefore, most of the standard metrics of Precision, such as Area Under the Sensitivity-Specficity Curve (AUC), Recall, and F1 score cannot be computed without assumptions on the distribution. To address that, we define the $RSP(\beta )$ score (Fig. 3A) as $(\beta \cdot RP -SP)/ (\beta \cdot RP+SP)$, where $\beta$ represents the importance of the RP vs. the SP. When $\beta =1$, there is an equal emphasis on RP and SP. In contrast, $\beta =0.05$ implies a willingness to forgo 20 RP to avoid 1 SP. This representation allows for tuning the type I and II errors of the analysis, in contrast with the permutation evaluation methods that focus solely on minimizing SP. The RSP of miMic is significantly higher $(p-value < 0.05)$ than the RSP of the SOTA models in 16S datasets and WGS datasets (Fig. 3B–C pink vs. all the other colors, the significance was tested for $\beta$ = 0.1, 0.5, and 1, see Additional file 1: Table S3).

Note that in additional datasets (not shown here) where the a priori nested ANOVA was not significant in any of the taxonomy levels, the post hoc test did not find any significant taxa. This emphasizes the importance of the a priori nested ANOVA to avoid useless taxa-specific tests.

Moreover, the cladogram structure is crucial. miMic obtains the best RSP scores when it starts on one of the 2 first taxonomy levels (kingdom or phylum, Fig. 3D-E). Interestingly, we find a clear positive correlation between the inner sisters’-label correlation (as defined in the “Methods” section) and the *RSP*(1) score (SCC = 0.588, *p*-value = 0.006, Fig. 3F). Only when there are practically no correlations between sisters is the RSP low. This finding underscores the importance of considering the correlation structure among taxa in the context of differential analysis. In contrast for high sister correlations, RSP is almost always 1 (Fig. 3B, C and Additional file 1: Fig. S2).

### miMic’s consistency and robustness

**Fig. 4:**
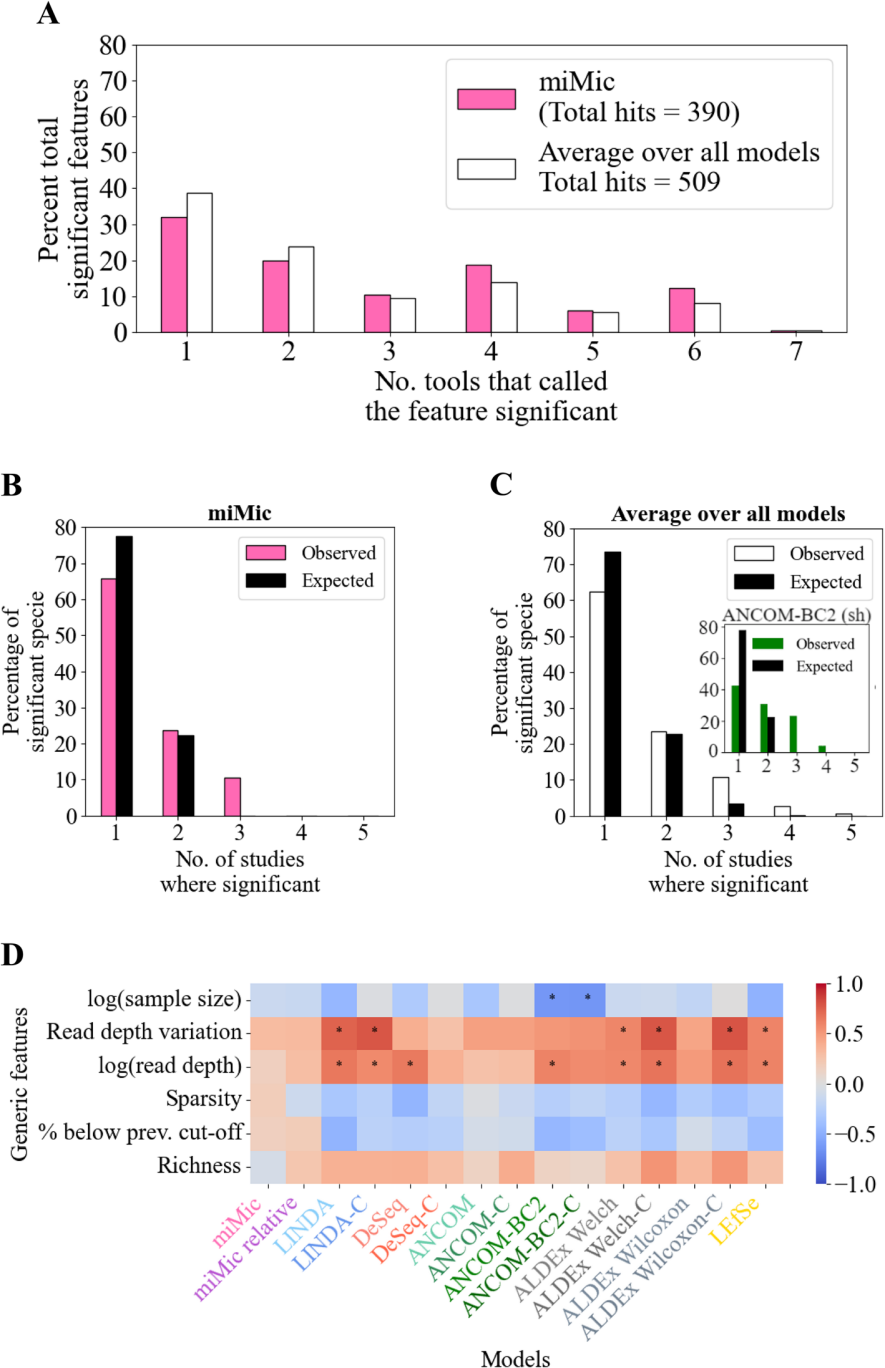
Consistency and robustness analysis of miMic. **A** Within-study differential abundance consistency analysis across multiple tools. The percentage of total significant features is plotted against the number of tools that identified the feature as significant. Results are shown for the miMic model (pink) and the average of all SOTA models (white). Refer to Additional file 1: Fig. S3 for a detailed analysis of all 13 tools. The total number of significant features identified by each tool is provided in the legend. miMic demonstrates slightly higher consistency compared to the average of all SOTA models. **B**–**C** Cross-study consistency analysis of differential abundance. The percentage of significant species is plotted against the number of studies where each species was identified as significant, conducted on five IBD cohorts. Results for miMic are depicted in pink (**B**), while those for the average SOTA model are shown in white (**C**). The expected results are presented in black (see the “Methods” section). Additionally, a parallel analysis on shuffled labels is provided for the ANCOM-BC2 model (green) within **C**. The models’ performance exceeds that of the expected random model. However, certain tools, such as ANCOM-BC2, exhibit artificially consistent results, as indicated in the inner plot of (**C**). For a comprehensive analysis of all 13 tools, refer to Supplementary Material Fig. S4. **D** Sensitivity robustness assessment. The heatmap illustrates the SCCs between each generic dataset characteristic and the percentage of significant taxa identified by each tool per dataset. Positive correlations are depicted in red, while negative correlations are shown in blue. Stars indicate a significant correlation (*p*-value < 0.05). miMic demonstrates robustness across all tested generic features in 16S datasets. For parallel analyses conducted on 16S and WGS cohorts, detailing the percentage of significant taxa identified by each tool per dataset and RSP score, refer to Supplementary Material Fig. S5

We next investigated the overlap in significant taxa across tools within each dataset [43]. While this is not an absolute measure of accuracy, consistency implies that the assumptions made in the analysis have a limited effect on the results. If two methods with different assumptions produce consistent results, one can assume that the results are not strongly affected by the assumptions. miMic is more consistent with other models than the average of all models (Fig. 4A and Additional file 1: Fig. S3 — where miMics’ distribution of overlap is more skewed to higher overlap values than the average overlap distribution of the other models).

We next investigated the consistency of miMic (vs. other models) across datasets of the same disease. We specifically focused on IBD as a phenotype, which has been shown to exhibit a strong effect on the microbiome and to be relatively reproducible across studies [44, 45]. We acquired five datasets for this analysis representing the microbiome of individuals with IBD compared with individuals without IBD (see the “Methods” section). We ran all differential abundance analysis tools on each individual dataset and restricted our analyses to the 81 species found across all datasets. Tools that generally identify more species as significant are accordingly more likely to identify species as consistently significant compared with tools with fewer significant hits. We thus compared the observed distribution against the expected distribution given random labels. miMic demonstrates notable consistency in findings across different cohorts, with over 10% of significant species consistent within three different cohorts (Fig. 4B). While other models show similar behavior on average, miMic stands out for its lack of false consistent species. Notably, all tools exhibit significantly higher consistency than random expectation across these datasets (Fig. 4B and C and Additional file 1: Fig. S4).

Another limitation of a statistical tool may be the effect of technical aspects of the sample on the fraction of real-label positives and shuffled positives in different datasets. We analyzed the correlation between multiple characteristics such as read depth, sparsity, richness, and dataset size and the percentage of significant taxa identified by each tool per dataset. In contrast with most other methods, we found no significant correlations of any of these factors with the miMics’ percentage of significant taxa (Fig. 4D). However, as expected, we do find in WGS correlations of the richness with the number of positive results (since there are more species tested — Additional file 1: Fig. S5).

### Example of miMic application and code availability

miMic is readily accessible through PyPi via https://pypi.org/project/mimic-da/ [46]. We here follow the utilization of miMic using an IBD cohort [47] as an illustrative example. The same example is given with detailed commands and formats in the Additional file 1. First, the ASVs undergo preprocessing via the MIPMLP pipeline employing default parameters (taxonomy level = 7, taxonomy group = Sub-PCA, normalization = log, epsilon = 0.1). Subsequently, 2D images are generated using iMic.

An a priori nested ANOVA test is executed, and the *p*-value for each taxonomy level test is presented until significance is identified. In cases where there are no taxonomy levels exhibiting a significant correlation with the tag, it implies a lack of discernible relationship between the microbiome data and the targeted label. In such cases, there is no point in advancing to the following stages. Upon detecting significance at any level in the previous step, the Mann-Whitney test is further applied. In contrast, if the ANOVA is significant, miMic performs the Mann-Whitney test along trajectories from the broadest significant level.

**Fig. 5:**
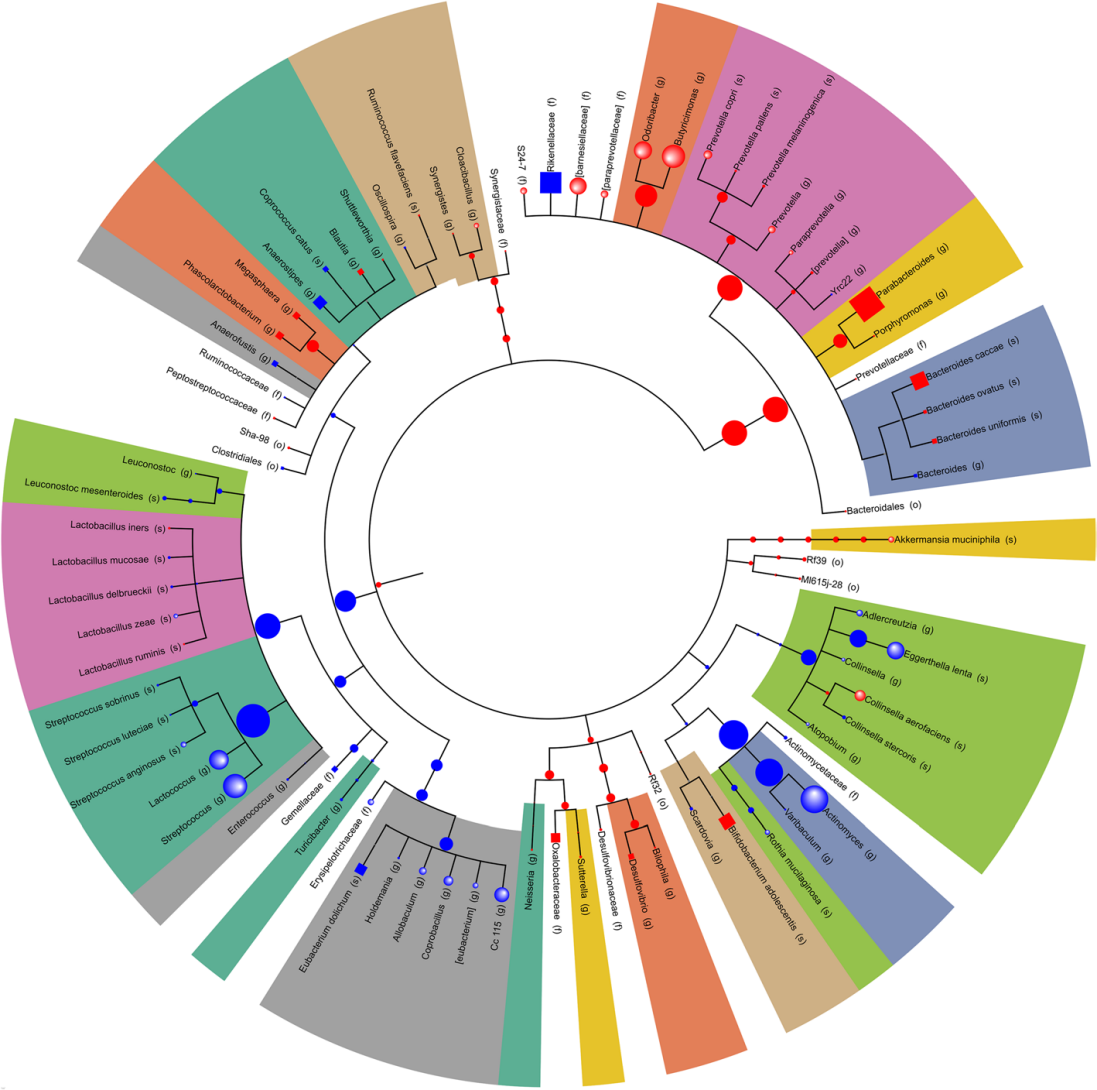
Differential abundance analysis results visualized on a cladogram for the IBD cohort. Each color represents the sign of the Mann-Whitney score (blue for positive scores, red for negative scores, and gray for non-significant taxa in internal nodes). The node size corresponds to -log10(*p*-value) from the Mann-Whitney test in miMic. The node shape represents its origin of significance: spheres were identified by both miMic and the Mann-Whiteny test on leaves, circles were identified by miMic only, and squares were identified by only the Mann-Whitney test. The colors represent the taxonomic family of each node

miMic offers a comprehensive suite of data visualization tools that provide a holistic perspective on the cohort’s differential analysis:

**Cladogram** (Fig. 5): This visual representation allows users to delve into significant Mann-Whitney scores and their associated *p*-values. It facilitates the understanding of complex relationships among taxa within the context of the label.

**Fig. 6:**
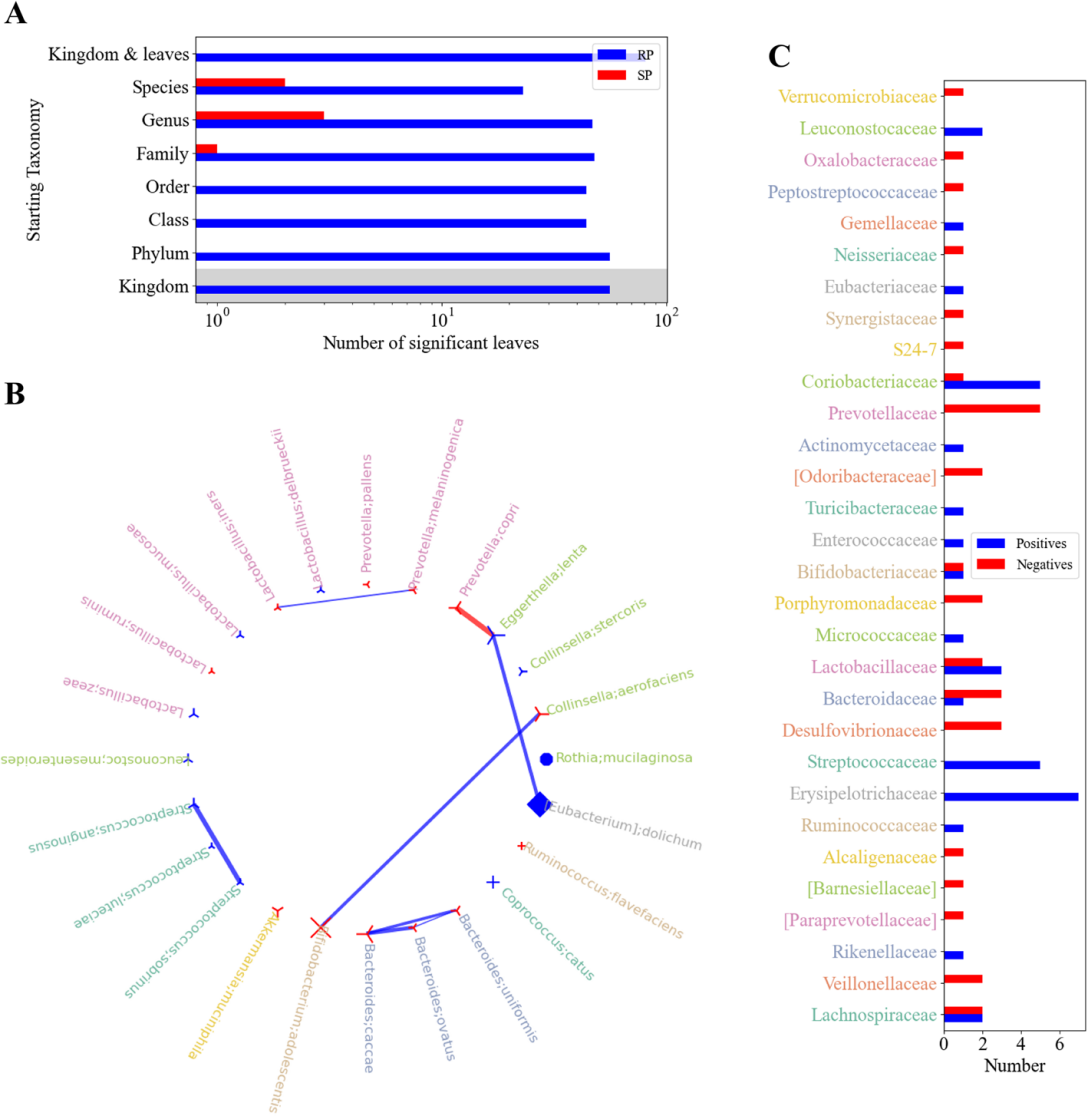
miMic’s plots - example on IBD cohort. **A** Bar plot illustrating the taxonomy levels in the miMic test vs. the number of significant findings in a real run (RP) shown in blue, and in a shuffled run (SP) shown in red. The highest bar plot represents the actual RP vs. SP of the selected taxonomy level of miMic combined with the leaves test as explained in the Methods. Taxonomy levels used for the a priori nested ANOVA test are shaded in gray. The number of RP significantly exceeds the number of SP. **B** Interaction between significant taxa found in miMic. Each taxon is colored according to its significant family color, similar to Fig. 5 above. Each node shape represents the taxon’s order. An edge is drawn between two nodes if their SCC is above 0.3 (user-adjustable) and its $p-value < 0.05$. The width of the edge corresponds to its SCC. A blue edge represents a positive relation, while a red edge represents a negative one. **C** Analysis of significant positive and negative relations within taxonomic families. The *y*-axis displays significant families in the cohort (defined by a family that has at least 1 significant descendant), while the *x*-axis shows the count of positive relations within a family in blue or the count of negative relations within a family in red. Each family is colored according to its color in the interaction network in **B** and the cladogram of correlations in Fig. 5 above

**Taxa analysis** (Fig. 6A): This plot unveils critical insights, including the counts of RP (blue bars) and SP (red bars) across various starting taxonomy levels of the trajectories of the Mann-Whitney test. Additionally, it provides important information on the significance of the ANOVA starting at each taxonomy level. Note the nested ANOVA stops once one of the taxonomy levels is significant. Therefore, the taxonomy levels used during the “test” mode have a gray background. Remarkably, many cohorts exhibit a scarcity of SP.

$\varvec{RSP(\beta )}$ s**cores** (The RSP plot of the IBD cohort is not shown here, see Supp Mat. Fig. S7): As mentioned $RSP(\beta )$ may differ among $\beta$ values. This plot shows the $\beta$ values where the RSP is high enough for the results to be trusted.

**Inner taxa interactions** (Fig. 6B): The correlation in expression between taxa, as defined by their Spearman correlation (only significant correlations are drawn). This shows a very strong association between the most significant microbes, highlighting that many of the reported associations may be the result of linkage with other microbes.

**Taxa-label relations** (Fig. 6C): This visual aid sheds light on both positive and negative relationships between labels and taxonomic families. It shows the families that have consistent associations with the label, while other families have some positive and some negative associations. For example, in the IBD cohort, the *Lachnospiraceae* family has varied relations to IBD with an equal number of significant positive associations and significant negative associations within the family. While *Strepctococcaceae* family is consistent with its positive associations with IBD.

**Taxa distributions over different taxonomy levels** Through the application of log Sub-PCA in the MIPMLP processing, taxa distributions exhibit a notable tendency toward normality. This transformation is particularly evident when examining taxonomic distributions across different levels. In the coarser taxonomy levels, such as kingdom and phylum, the distributions approach a normal distribution for broad levels (Additional file 1: Fig. S6).

## Discussion

The host microbiome has been associated with a myriad of phenotypes. This association is often performed using three main arguments: A) Samples with a given condition are closer one to each other than to samples without this label [48, 49], B) the microbiome samples can be used to predict the phenotye using machine learning methods [42, 50, 51], or C) specific taxa are associated with a condition/property/label of the host [11, 12, 14, 17]. This last approach has been termed differential abundance analysis, and a large number of methods have been proposed to perform it. Differential abundance analysis suffers from three inherent extensively discussed limitations: a large number of taxa vs. a small number of samples in typical experiments, very limited overlap between the taxa in different samples, and non-normal distribution of the taxa frequencies (even after log-normalization). An additional less discussed limitation is the correlation between the expression level of taxonomically related taxa. We have here presented miMic that addresses these limitations by adding to the standard multiple measurement correction, and alternative that taxa along the trajectory on the cladogram are associated with the label. We show that this ensures a high probability of finding real association, but keeps the probability of random associations low. This is the result of the typical positive correlations between sister taxa. Since miMic tests either observed taxa or taxa consistently significant along the cladogram, the main associations missed by it are rare microbe associations at coarse taxonomy levels. For example, assume two sister genera with precise associations with a label. Their mother taxon will not be detected by miMic. However, such cases are rare, since 1) Most sisters’ frequencies are positively and not negatively correlated. 2) Even in the case of opposite associations, two opposing associations will cancel one another only if they are of the same order. Indeed, in the example presented here, we show the detection of such opposite associations. Moreover, to address that we propose to start miMic at different levels. However, consistently the phylum level or the kingdom level gave the highest RSP scores.

However, the main limitations of miMic emerge from the inherent analysis of microbiome samples that have a very limited overlap between datasets. Indeed, the fraction of taxa overlapping between any two datasets is typically less than 0.1 [52]. Thus, differential expression analysis in per-definition is often not well transferred between datasets. However, the requirement of consistent associations along the cladogram trajectory ensures that at least at a coarse taxonomical description, such association can be maintained.

To compare different methods with no GT, we developed a novel measure denoted RSP, this measure represents the difference between the number of detected associations (or any other significance test) in real and shuffled samples, where the number of real associations is multiplied by $0<\beta <1$. If the number of real associations is 20 times larger than random associations $RSP(0.05)=0$. While in miMic RSP is high and close to 1 even for $\beta =0$, most current methods, have low and often negative values even for $\beta =0.2$. Note that the RSP measure can be useful for association tests, with no GT, such as genetic associations [53–55].

miMic is accessible as a Python package, https://pypi.org/project/mimic-da/, available online athttps://github.com/oshritshtossel/miMic as well as a website https://micrOS.math.biu.ac.il [46, 56, 57].

## Conclusions

Microbiome data is often very high dimensional, and as such requires multiple measurement problem for statistical tests on each microbe. However, the taxonomic structure can be used to mitigate this limitation and ensure a high discovery rate with a very low false discovery rate. Moreover, it allows for an a priori test if the microbiota is related to a given label. These two concepts as integrated in miMic (https://pypi.org/project/mimic-da/ and https://github.com/oshritshtossel/miMic) [46, 56] by an intuitive visualization allow for rapid and accurate statistical tests of microbiome-condition association. This can be tested by the newly proposed weighted RSP score to compare real and shuffled positive fractions ratios.

Using the inherent structure of the data to reduce the effect of multiple measurements may be used in other biological contexts, such as gene expression, and pathway analysis.

## Methods

### Data

The different methods were tested both on 16S datasets (12 cohorts) and WGS datasets (8 cohorts). For more information about the datasets, see Additional file 1: Table S2.

#### Preprocessing

We preprocessed the 16S rRNA gene sequences (some downloaded by YAMAS https://github.com/YarinBekor/YaMAS [5]) and the shotgun metagenomics of each dataset using the MIPMLP pipeline [41]. The preprocessing of MIPMLP contains 4 stages: merging similar features based on the taxonomy, scaling the distribution, standardization to z-scores, and dimension reduction. For the miMic analysis, we merged the features at the species taxonomy by Sub-PCA. We performed log normalization on the patients. For all the other analyses, we followed the preprocessing reported in the manuscript (see Additional file 1: Table S4). No dimension reduction was used.

**Sub-PCA merging in MIPMLP.** A taxonomic level (e.g., species) is set. All the ASVs that are consistent with this taxonomy are grouped. A PCA (principal component analysis) is performed on this group. The components that explain more than half of the variance are added to the new input table. This was applied to the data analyzed by miMic.

**Log normalization in MIPMLP.** We logged (10 base) scale the features element-wise, according to the following formula:

1 $$\begin{aligned} x_{i,j} \rightarrow \log (x_{i,j}+\epsilon ), \end{aligned}$$

where $\epsilon$ is a minimal value $(= 0.1)$ to prevent log of zero values. This was applied to the data analyzed by miMic.

### miMic algorithm

The miMic algorithm contains 3 steps:

#### Data processing — generating the cladograms

The MIPMLP-processed ASVs vector is translated into a cladogram of means, such that the observed taxa are positioned in the leaves (with no sons) of the cladogram, and set their value to the preprocessed frequency to each leaf. Each internal node is the average of its direct descendants (Fig. 1D data processing).

#### A priori test — nested ANOVA

To test whether the dataset label distinguishes between the microbiome variances, we apply an a priori test that considers the cladogram structure, nested ANOVA. First, a regular ANOVA is applied on the kingdom level of all the cladograms. If the ANOVA is significant $(p-value < 0.05)$, then the whole test is defined as significant. If the first layer test is not significant, then a two-level nested ANOVA test is applied. If it is significant, the whole test is defined as significant, if not, we keep with the k-level nested ANOVA test iteratively till the leaves of the cladogram. We move to the post hoc Mann-Whitney test only if the a priori nested ANOVA is significant (Fig. 1D a priori nested ANOVA).

#### Post hoc — Mann-Whitney test

After the a priori test the Mann-Whitney test is applied. A Mann-Whitney test is applied to the first taxonomy in the cladograms if significant after multiple measurement corrections (Bonferroni or Benjamini Hochberg) we iteratively keep applying the Mann-Whitney test along the trajectories in the cladogram. A sister multiple measurement correction is applied once the mother is significant and the daughter is significant to all the sisters apart from the most significant one. Significant taxa along the whole trajectory are returned (Fig. 1D post hoc Mann-Whitney test 1). Beyond the taxa predicted along a path, miMic detects significant leaves identified through the Mann-Whitney test, with multiple measurement correction utilizing a Bonferroni adjustment, even in cases where they lack a significant ancestor (Fig. 1D post hoc Mann-Whitney test 2).

### Simulations setup

#### Generic hiererachial simulations

To illustrate the hierarchical dynamics, the simulations featured three sister taxa sharing a common mother with the average of their abundances. As assigned in the “Results” section, each daughter is assigned with $z_i$ and their mother is assigned with *m*, such that $m = (z_1+z_2+z_3)/3$. 2N samples are assumed where *N* are positive and *N* are negative. Three different scenarios were tested:

- **Simulation(0,0,0)** $$z1,z2,z3\sim \mathcal {N}(0,\,1)$$.
- **Simulation(0,0,**$$\varvec{\mu }$$**)** $$z1\sim \mathcal {N}(\mu ,\,1)$$ and $$z2,z3\sim \mathcal {N}(0,\,1)$$.
- **Simulation(0,**$$\varvec{\mu }$$**,**$$\varvec{\alpha \cdot \mu }$$**)** $$z1\sim \mathcal {N}(\mu ,\,1)$$, $$z2\sim \mathcal {N}(\alpha \cdot \mu ,\,1)$$ and $$z3\sim \mathcal {N}(0,\,1)$$.

The simulations are tested within different *N*s in the range of 20 to 1280, and different $\mu$s in the range of 0 and 1.

#### Microbiome-oriented simulations

We conducted microbiome-oriented simulations using three microbial datasets (PRJNA353587, *n *= 83; IBD, *n *= 257; ERP020401, *n *= 684).

We randomly selected 10 taxa along with all their raw ASVs and increased their abundances by 20% in samples randomly labeled as positive. A parallel process was executed for another set of 10 taxa in samples randomly labeled as negative.

### Evaluation methods and statistics

#### Sister correlation test

To evaluate the hypothesis that sister taxa tend to similarly relate to labels in comparison to random taxa, we define the following value for each taxon *j*:

2 $$\begin{aligned} s_j = \frac{|M0_j-M1_j|}{|V0_j-V1_j|^{0.5}}, \end{aligned}$$

where $M0_j$ is defined as the average of taxon *j* over all the samples lacking the label, such that $y_i = 0$, $M1_j$ is defined as the average of taxon *j* over all the samples having the label, such that $y_i = 1$,$V0_j$ is defined as the variance of taxon *j* over all the samples lacking the label, such that $y_i = 0$, $V1_j$ and is defined as the average of taxon *j* over all the samples having the label, such that $y_i = 1$. Then the SCC between sister taxa at different taxonomy levels (share the same mother in the cladogram) is calculated and the *p*-value is reported as well.

#### TP rate (TPR)

TPR is the probability that an actual positive will test positive. The TP is calculated only for the analysis of the simulations where the ground truth is known.

#### FP rate (FPR)

FPR is the probability that an actual negative will test positive. The FP is calculated only for the analysis of the simulations where the ground truth is known.

#### F1 score

To test the ratio between the TP and FP, we computed the F1 score of the microbiome-oriented simulations [58].

#### RSP score

Since in real datasets, no clear GT is defined, we define the term “real positives” (RP), by counting the number of significant taxa received by the test on the real dataset and the real labels, and the term “shuffled positives” (SP), by counting the number of significant taxa received by the test on shuffled labels. We further define the $RSP(\beta )$ score as $(\beta \cdot RP -SP)/(\beta \cdot RP+SP)$, where $\beta$ represents the confidence. By changing the $\beta$ values one can control the importance ratio between the RP and SP.

#### Within-study differential abundance consistency analysis across multiple tools

In our cross-model consistency analysis of differential abundance [43], we assessed the agreement among different tools across all datasets by aggregating all ASVs identified as significant by at least one tool in the 12 16S datasets studied here. The number of tools that identified each ASV as differentially abundant was then tabulated. Given that some models had multiple variants, such as variations with or without multiple measurement correction or different statistical tests within ALDEx2, we consolidated the methods into seven distinct models: ANCOM (encompassing ANCOM and ANCOM-C), ANCOM-BC2 (comprising ANCOM-BC2 and ANCOM-BC2-C), DeSeq (including DeSeq and DeSeq-C), ALDEx2 (encompassing ALDEx2 Welch, ALDEx2 Welch-C, ALDEx2 Wilcoxon, and ALDEx2 Wilcoxon-C), miMic (miMic and miMic relative), and LEfSe. A taxon was defined as significant in a group if it was detected as significant with $p<0.05$ by at least one of its variants. We calculated the average percentage of total significant features across all SOTA models for comparative purposes.

#### Cross-study consistency analysis of differential abundance

We used 5 different IBD cohorts (IBD, ERP021216, OK94, PRJNA353587, and PRJNA419097). We collapsed all feature abundances to the species level. Note that taxonomic classification was performed using several different methods, which represents another source of variation. We then ran all differential abundance tools on these datasets to associate taxa with IBD. For each tool and study combination, we determined which species were significantly different with a *p*-value lower than 0.05 (where relevant). For each tool, we then tallied the number of times each species was significant, i.e., how many datasets each species was significant in based on a given tool. The null expectation distributions of these counts per tool were generated by randomly sampling species from each dataset. The probability of sampling a species (i.e., calling it significant) was set to be equal to the proportion of actual significant species in this dataset in this method. This procedure was repeated 1000 times, with species replicates equal to the actual number of tested species (81). For each replicate, we tallied the number of times the species was sampled across datasets. Note that to simplify this analysis, we ignored the directionality of the significance (e.g., whether it was higher in case or control samples). For ANCOM-BC2, we repeated the analysis with shuffled labels to show the high consistency is simply the result of a very high fraction of SP taxa.

#### Robustness analysis

To assess the robustness of each tool across various generic dataset characteristics, including sparsity, read depth (variance and median), mean sample ASV richness, cutoff, and dataset size, we calculated the Spearman correlation between each tool’s performance metrics and these attributes. Tool performance was evaluated based on either the percentage of significant ASVs or the RSP(1) score. We visualized the SCCs using a heatmap, with significant correlations ($p < 0.05$) annotated with a star.

### Statistical tests

Each method underwent 10 repeated shuffling (in a shuffled configuration) on every dataset. Subsequently, the average $RSP(\beta )$ score was computed for various values of $\beta$. To evaluate the significance of the models and their interactions, a two-way ANOVA test was conducted for typical $\beta$ of (0.05, 0.1, 0.5, 1) separately. This ANOVA included the dataset and model as factors. For the $\beta$ values, where the model was significant, a one-sided *t*-test was subsequently employed to compare the two best models (defined by their RSP score).

### Supplementary Information

**Additional file 1.** Includes supplementary figures (Figures S1 - S7), supplementary tables (Tables S1 - S4), as well as technical documentation detailing the steps for utilizing miMic through PyPi or its website interface.

**Additional file 2.** Contains the review history.

## Data Availability

All data used in this study are sourced from published manuscripts, with the origins of each dataset detailed in Additional file 1: Table S2 [47, 59–74]. Some datasets were retrieved from the NCBI (National Center for Biotechnology Information) under the following SRA numbers: ERP021216 [75], ERP020401 [62], PRJNA353587 [69], PRJNA412501 [70], and PRJNA419097 [71], and processed using YAMAS [5]. miMic is accessible as a Python package, which can be found at https://pypi.org/project/mimic-da/ [46], with its source code hosted under GNU Lesser General Public License on GitHub at https://github.com/oshritshtossel/miMic Version v.0.0 of the code is also archived on Zenodo at https://zenodo.org/doi/10.5281/zenodo.10958274 [56, 76]. Additionally, a web interface for miMic is available at https://micrOS.math.biu.ac.il/ [57]. The complete analysis code, inclusive of statistical analyses, comparisons, and visualizations, can be accessed under GNU Lesser General Public License on GitHub at https://github.com/oshritshtossel/miMic_all_analyses. Version v.0.0 of the code is also archived on Zenodo at https://zenodo.org/doi/10.5281/zenodo.10952811 [77, 78].

## Appendix: theoretical calculations

### Joint distribution

Assume $z_i$ for node *i* value and $m=\sum _i z_i/3$.

### Case A - $z1,z2,z3\sim \mathcal {N}(0,\,1)$

3 $$\begin{aligned} M=P(z1-significant,m-significant)=\int _{k/\sqrt{(}3)}df \int _k dz p_N((z1+z2+z3)/3=m,z1=z) \end{aligned}$$

where *k* is the cutoff for a $\alpha$ uncorrected *p* value.

4 $$\begin{aligned} g(f,z1)=p_N((z1+z2+z3)/3=m,z1=z)=p_N(z1+z2+z3=3*m,z1=z)=p_N(z3+z2=3*m-z,z1=z) \end{aligned}$$

5 $$\begin{aligned} =p_N(z3+z2=3*m-z)p(z1=z)=N(3*m-z,0,2)N(z,0,1) \end{aligned}$$

6 $$\begin{aligned} M=\int _k dz N(z,0,1)\int _{k/\sqrt{(}3)}df N(3*m-z,0,2) \end{aligned}$$

Change variables to $u=3*m-z$

—7 $$\begin{aligned} M=\frac{1}{3}\int _k dz N(z,0,1)\int _{\sqrt{3}k-z}du N(u,0,2) \end{aligned}$$

We can again replace the variable to $u1=u/\sqrt{2}$.

8 $$\begin{aligned} M=\frac{\sqrt{2}}{3}\int _k dz N(z,0,1)\int _{\frac{\sqrt{3}k-z}{\sqrt{2}}}du1 N(u1,0,1)=\frac{\sqrt{2}}{3}\int _k dz N(z,0,1)*\left( 1-\Phi \left( \frac{\sqrt{3}k-z)}{\sqrt{2}}\right) \right. \end{aligned}$$

9 $$\begin{aligned} M=\frac{\sqrt{2}}{3}\int _k dz N(z,0,1)* \left( \Phi \left( \frac{z-\sqrt{3}k}{\sqrt{2}}\right) \right. \end{aligned}$$

### Case B - $z1\sim \mathcal {N}(\mu ,\,1)$ and $z2,z3\sim \mathcal {N}(0,\,1)$

10 $$\begin{aligned} M=P(z1-significant,m-significant)=\int _{k/\sqrt{3}}df \int _k dz p_N((z1+z2+z3)/3=m,z1=z) \end{aligned}$$

where *k* is the cutoff for a $\alpha$ uncorrected *p* value.

11 $$\begin{aligned} g(f,z1)=p_N((z1+z2+z3)/3=m,z1=z)=p_N(z1+z2+z3=3*m,z1=z)=p_N(z3+z2=3*m-z,z1=z) \end{aligned}$$

12 $$\begin{aligned} =p_N(z3+z2=3*m-z)p(z1=z)=N(3*m-z,0,2)N(z,\mu ,1) \end{aligned}$$

13 $$\begin{aligned} M=\int _k dz N(z,\mu ,1)\int _{k/\sqrt{3}}df N(3*m-z,0,2) \end{aligned}$$

Change variables to $u=3*m-z$

14 $$\begin{aligned} M=\frac{1}{3}\int _k dz N(z,\mu ,1)\int _{\sqrt{3}k-z}du N(u,0,2) \end{aligned}$$

We can again replace the variable to $u1=u/\sqrt{2}$.

15 $$\begin{aligned} M=\frac{\sqrt{2}}{3}\int _k dz N(z,\mu ,1)\int _{\frac{\sqrt{3}k-z}{\sqrt{2}}}du1 N(u1,0,1)=\frac{\sqrt{2}}{3}\int _k dz N(z,\mu ,1)* \left( 1 - \Phi \left( \frac{\sqrt{3}k-z}{\sqrt{2}}\right) \right) \end{aligned}$$

16 $$\begin{aligned} M=\frac{\sqrt{2}}{3}\int _k dz N(z,\mu ,1)*\Phi \left. \left( \frac{z-\sqrt{3}k}{\sqrt{2}}\right) \right) \end{aligned}$$

### Case C - $z1,z2\sim \mathcal {N}(\mu ,\,1)$ and $z3\sim \mathcal {N}(0,\,1)$, such that $\alpha$ is 1

17 $$\begin{aligned} M=P(z1-significant,m-significant)=\int _{k/\sqrt{3}}df \int _k dz p_N((z1+z2+z3)/3=m,z1=z) \end{aligned}$$

where *k* is the cutoff for a $\alpha$ uncorrected *p* value.

18 $$\begin{aligned} g(f,z1)=p_N((z1+z2+z3)/3=m,z1=z)=p_N(z1+z2+z3=3*m,z1=z)=p_N(z3+z2=3*m-z,z1=z) \end{aligned}$$

19 $$\begin{aligned} =p_N(z3+z2=3*m-z)p(z1=z)=N(3*m-z,\mu ,2)N(z,\mu ,1) \end{aligned}$$

20 $$\begin{aligned} M=\int _k dz N(z,\mu ,1)\int _{k/\sqrt{3}}df N(3*m-z,\mu ,2) \end{aligned}$$

Change variables to $u=3*m-z$

21 $$\begin{aligned} M=\frac{1}{3}\int _k dz N(z,\mu ,1)\int _{\sqrt{3}k-z}du N(u,\mu ,2) \end{aligned}$$

We can again replace the variable to $u1=(u -\mu )/\sqrt{2}$.

22 $$\begin{aligned} M=\frac{\sqrt{2}}{3}\int _k dz N(z,\mu ,1)\int _{\frac{\sqrt{3}k-z-\mu }{\sqrt{2}}}du1 N(u1,0,1)=\frac{\sqrt{2}}{3}\int _k dz N(z,\mu ,1)* \left( 1-\Phi \left( \frac{\sqrt{3}k-z-\mu }{\sqrt{2}}\right) \right) \end{aligned}$$

23 $$\begin{aligned} M=\frac{\sqrt{2}}{3}\int _k dz N(z,\mu ,1)* \left( \Phi \left( \frac{\sqrt{3}k-z-\mu }{\sqrt{2}}\right) \right) \end{aligned}$$
