# Supplementary material for: mi-Mic: a novel multi-layer statistical test for microbiota-disease associations
Source: Genome Biol. 2024 May 1;25:113. doi: 10.1186/s13059-024-03256-0 (PMC11064322; doi:10.1186/s13059-024-03256-0)
Supplement: Supplementary file 1 — Additional file 1. Includes supplementary figures (Figures S1 - S7), supplementary tables (Tables S1 - S4), as well as technical documentation detailing the steps for utilizing miMic through PyPi or its website interface. [file 13059_2024_3256_MOESM1_ESM.pdf]

Additional File 1

Oshrit Shtossel[1], Shani Finkelstein[1], and Yoram Louzoun[1,*]

[1]Department of Mathematics, Bar-Ilan University, Ramat Gan 52900, Israel
[*]Corresponding author: Yoram Louzoun, louzouy@math.biu.ac.il

April 11, 2024

1 How to apply miMic via PyPi - Running Example

1.1 Install the package

```
pip install mimic-da
```

1.2 How to apply miMic

See 'example use.py' for an example of how to use miMic. The example contains the following steps:

1. Import miMic and additional packages.

```
from mimic_da import apply_mimic
import pandas as pd
```

2. Load the raw ASVs table in the following format:
   - The first column is named "ID".
   - Each row represents a sample and each column represents an ASV.
   - The last row contains the taxonomy information, named "taxonomy".

   ```
   df = pd.read_csv("example_data/for_process.csv")
   ```

   - Note: 'for process.csv' is a file that contains the raw ASVs table in the required format, you can find an example file in the 'example data' folder in GitHub.

3. Load a tag table as csv, such that the tag column is named "Tag".

   ```
   tag = pd.read_csv("example_data/tag.csv",index_col=0)
   ```

   - Note: 'tag.csv' is a file that contains the tag table in the required format, you can find an example tag in the 'example data' folder in GitHub.

4. Specify a folder to save the output of the miMic test.

```
folder = "example_data/2D_images"
```

- Note: '2D images' is a folder that will be created in your current working directory, and the output of the miMic test will be saved there.

5. Apply MIPMLP.

- MIPMLP using defaulting parameters, you can find more in 'Note' section below.
- taxonomy_group: ["sub PCA", "mean", "sum"], "sub PCA" method is preferred.

```
processed = apply_mimic(folder=folder, tag=tag, mode="preprocess",
          preprocess=True, rawData=df,taxnomy_group='sub_PCA')
```

- Note: MIPMLP is a package that is used to preprocess the raw ASVs table, see MIPMLP PyPi https://pypi.org/project/MIPMLP/ or MIPMLP website https://mip-mlp.math.biu.ac.il/Home for more explanations. If you have your own processed data, set preprocess to False, and use your processed data as input for processed parameter in the next step.

6. Apply miMic test. miMic using the following hyperparameters:

- **eval:** evaluation method, ["man", "corr", "cat"]. The default is "man".
  - "man" for binary labels.
  - "corr" for continuous labels.
  - "cat" for categorical labels.
- **sis:** apply sister correction,["fdr_bh", "bonferroni", "no"]. The default is "fdr_bh".
- **correct_first:** apply FDR correction to the starting taxonomy level according to sis parameter,[True, False]. The default is True.
- **mode:** 2 different formats of running,["test", "plot"]. The default is "test".
- **save:** whether to save the corrs_df of the miMic test to computer,[True, False]. The default is True.
- **tax:** starting taxonomy of the post hoc test,["None", 1, 2, 3, "noAnova", "nosignifacnt"].
  - In "test" mode the defaulting value is "None".
  - In the "plot" mode the tax is set automatically to the selected taxonomy of the "test" mode [1, 2, 3, "noAnova"].
  - "noAnova", where a priori nested ANOVA test is not significant.
  - "nosignificant", where a priori nested ANOVA test is not significant and miMic did not find any significant taxa in the leafs. In this case, the post hoc test will not be applied.
- **colorful:** Determines whether to apply colorful mode on the plots [True, False]. The default is True.
- **threshold_p:** the threshold for significant values. The default is 0.05.
- **THRESHOLD_edge:** the threshold for having an edge in the "interaction" plot. The default is 0.5.
- **processed:** the processed data from the previous step. The default is None.

- **apply_samba:** whether to apply SAMBA or not. The default is True (Boolean).
- **samba_output:** if you already have samba outputs- miMic will read it from the folder you specified, else miMic will apply samba and set samba_output to None.

```
    if processed is not None:
   taxonomy_selected ,samba_output = apply_mimic(folder , tag , eval="man" ,
   if taxonomy_selected is not None:
       apply_mimic(folder , tag , mode="plot" , tax=taxonomy_selected ,
              eval="man" , sis='fdr_bh' , samba_output=samba_output ,
save=False , threshold_p=0.05 , THRESHOLD_edge=0.5)
```

7. Note: if apply_samba is set to True, miMic will apply samba-metric. If save is set to True, the output will be saved to the folder you specified. See SAMBA PyPi https://pypi.org/project/samba-metric/ for more explanations.

1.3 miMic output

miMic will output the following:

- If save is set to True, SAMBA's outputs and the following 'csv' will be saved to your specified folder:
  - **corrs_df:** a data frame containing the results of the miMic test (including Utest results).
  - **just_mimic:** a data frame containing the results of the miMic test without the Utest results.
  - **u_test_without_mimic:** a data frame containing the results of the Utest without the miMic results.
  - **miMic&Utest:** a data frame containing the joint results of miMic and Utest tests.
- If mode is set to "plot", plots will be saved in the folder named 'plots' in your current working directory. The following plots will be saved:
  1. **tax_vs_rp_sp_anova_p:** Bar plot illustrating the taxonomy levels in the miMic test vs. the number of significant findings in a real run (RP) shown in blue, and in a shuffled run (SP) shown in red. The highest bar plot represents the actual RP vs. SP of the selected taxonomy level of miMic combined with the leaves test as explained in the Methods. Taxonomy levels used for the a priori nested ANOVA test are shaded in grey. The number of RP significantly exceeds the number of SP (see Fig. 6 A).
  2. **rsp_vs_beta:** Representation of RSP(β) score as a function of the confidence level beta. An RSP score of 1 indicates the presence of only RP without any SP (see Supp. Mat. Fig. S7).
  3. hist: Histogram of the distribution of logged abundances within each level of taxonomy on the cladogram of means. Different line styles and line weights are assigned to each taxonomy level for distinction (see Supp. Mat. Fig. S6).
  4. **corrs_within_family** Analysis of significant positive and negative relations within taxonomic families. The y-axis displays significant families in the cohort (defined by a family that has at least 1 significant descendant), while the x-axis shows the count of positive relations within a family in blue or the count of negative relations within a

family in red. Each family is colored according to its color in the interaction network in Fig. 6 **(B)** and the cladogram of correlations in Fig. 5 (see Fig. 6 C).

5. **interaction:** Interaction between significant taxa found in miMic. Each taxon is colored according to its significant family color, similar to Fig. 5 above. Each node shape represents the taxon's order. An edge is drawn between two nodes if their Spearman correlation coefficient (SCC) is above 0.3 (user-adjustable) and its p-value ¡ 0.05. The width of the edge corresponds to its SCC. A blue edge represents a positive relation, while a red edge represents a negative one (see Fig. 6 B).
6. **correlations_tree:** Differential abundance analysis results are visualized on a cladogram for the IBD cohort. Each color represents the sign of the Mann-Whitney score (blue for positive scores, red for negative scores, and grey for non-significant taxa). The node size corresponds to -log10(p-value) from the Mann-Whitney test in miMic. The node shape represents its origin of significance: spheres were identified by both miMic and the Mann-Whiteny test on leaves, circles were identified by miMic only, and squares were identified by only the Mann-Whitney test. The colors represent the taxonomic family of each node (see Fig. 5).

2 How to apply miMic via the micrOS website -Running Example

2.1 Get into micrOS

Entering the following URL https://micros.math.biu.ac.il.

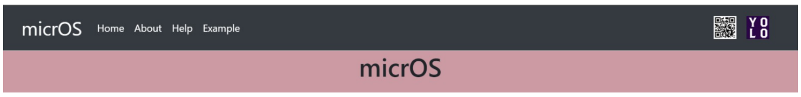

2.2 How to apply miMic

1. Load the raw ASVs table via the "Select OTU table" button (see below) in the following format:
   - The first column is named "ID".
   - Each row represents a sample and each column represents an ASV.
   - The last row contains the taxonomy information, named "taxonomy".
2. Load a tag table as csv via the "Select tag file" button (see below), such that the tag column is named "Tag".

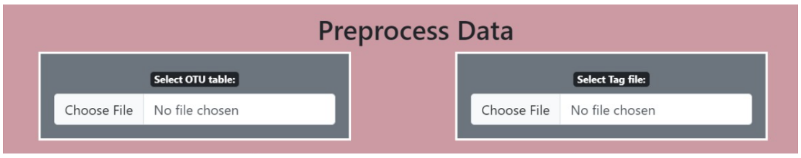

3. Tick in V the miMic option (see below).

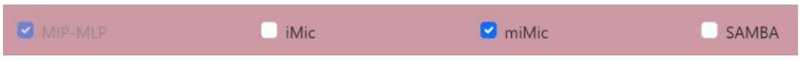

4. Choose the running parameters (see image below). For explanations about each parameter see How to apply miMic via PyPi - Running Example how to apply miMic 6.

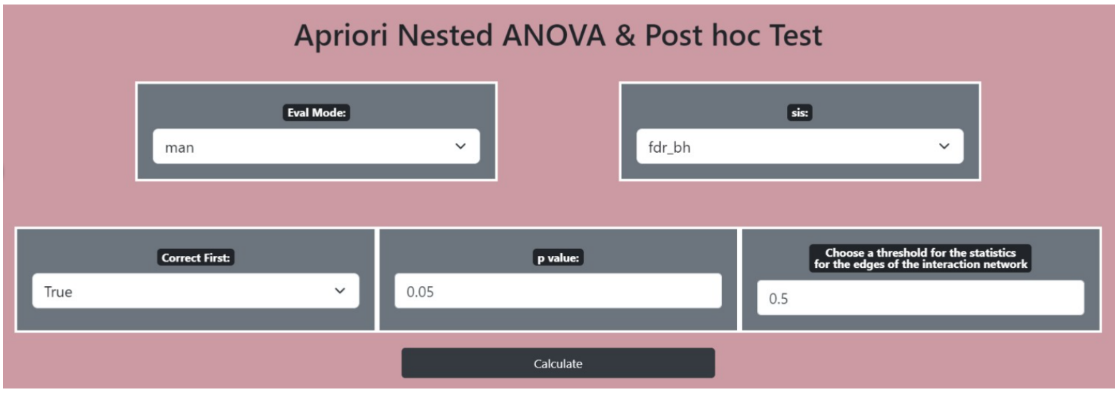

2.3 miMic output

miMic will output the following:

- **corrs_df:** a data frame containing the results of the miMic test (including Utest results).
- The following plots will be presented:

  1. **tax_vs_rp_sp_anova_p:** Bar plot illustrating the taxonomy levels in the miMic test vs. the number of significant findings in a real run (RP) shown in blue, and in a shuffled run (SP) shown in red. The highest bar plot represents the actual RP vs. SP of the selected taxonomy level of miMic combined with the leaves test as explained in the Methods. Taxonomy levels used for the a priori nested ANOVA test are shaded in grey. The number of RP significantly exceeds the number of SP (see Fig. 6 A).
  2. **rsp_vs_beta:** Representation of RSP(β) score as a function of the confidence level beta. An RSP score of 1 indicates the presence of only RP without any SP (see Supp. Mat. Fig. S7).
  3. hist: Histogram of the distribution of logged abundances within each level of taxonomy on the cladogram of means. Different line styles and line weights are assigned to each taxonomy level for distinction (see Supp. Mat. Fig. S6).
  4. **corrs_within_family** Analysis of significant positive and negative relations within taxonomic families. The y-axis displays significant families in the cohort (defined by a family that has at least 1 significant descendant), while the x-axis shows the count of positive relations within a family in blue or the count of negative relations within a family in red. Each family is colored according to its color in the interaction network in Fig. 6 **(B)** and the cladogram of correlations in Fig. 5 (see Fig. 6 C).
  5. **interaction:** Interaction between significant taxa found in miMic. Each taxon is colored according to its significant family color, similar to Fig. 5 above. Each node

shape represents the taxon's order. An edge is drawn between two nodes if their Spearman correlation coefficient (SCC) is above 0.3 (user-adjustable) and its p-value ¡ 0.05. The width of the edge corresponds to its SCC. A blue edge represents a positive relation, while a red edge represents a negative one (see Fig. 6 B).

6. **correlations_tree:** Differential abundance analysis results are visualized on a cladogram for the IBD cohort. Each color represents the sign of the Mann-Whitney score (blue for positive scores, red for negative scores, and grey for non-significant taxa). The node size corresponds to -log10(p-value) from the Mann-Whitney test in miMic. The node shape represents its origin of significance: spheres were identified by both miMic and the Mann-Whiteny test on leaves, circles were identified by miMic only, and squares were identified by only the Mann-Whitney test. The colors represent the taxonomic family of each node (see Fig. 5).

One should click on each plot to download it into the computer.

3 Supplementary Figures

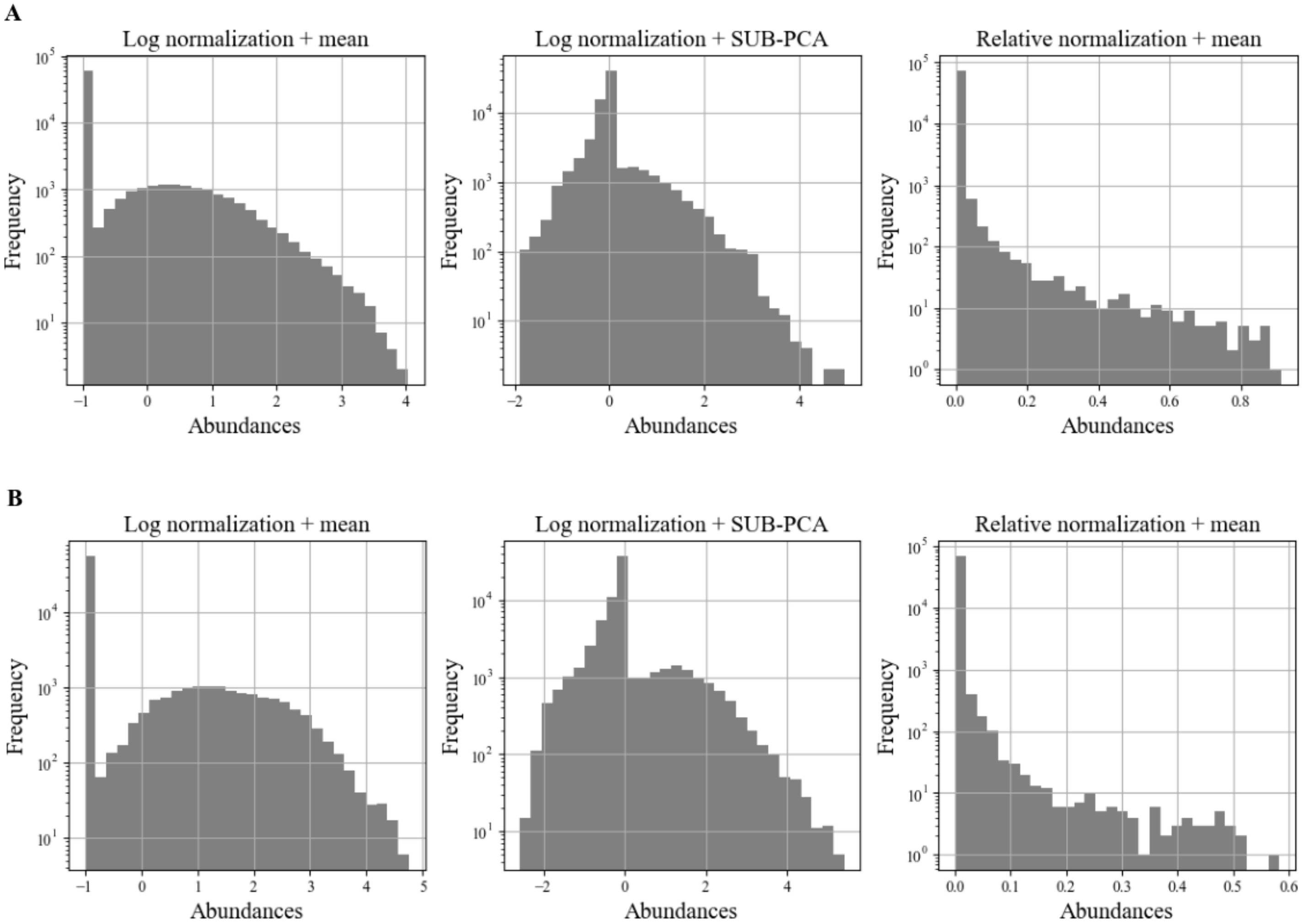

Fig. S1: Microbiome distributions over 2 IBD cohorts (referred to as IBD **A-C** and Jacob **D-F** over different preprocessing (grouping and normalization), such as mean grouping + log normalization **A, D**, Sub-PCA grouping + log normalization **B, E** and mean grouping + relative normalization **C, F**.

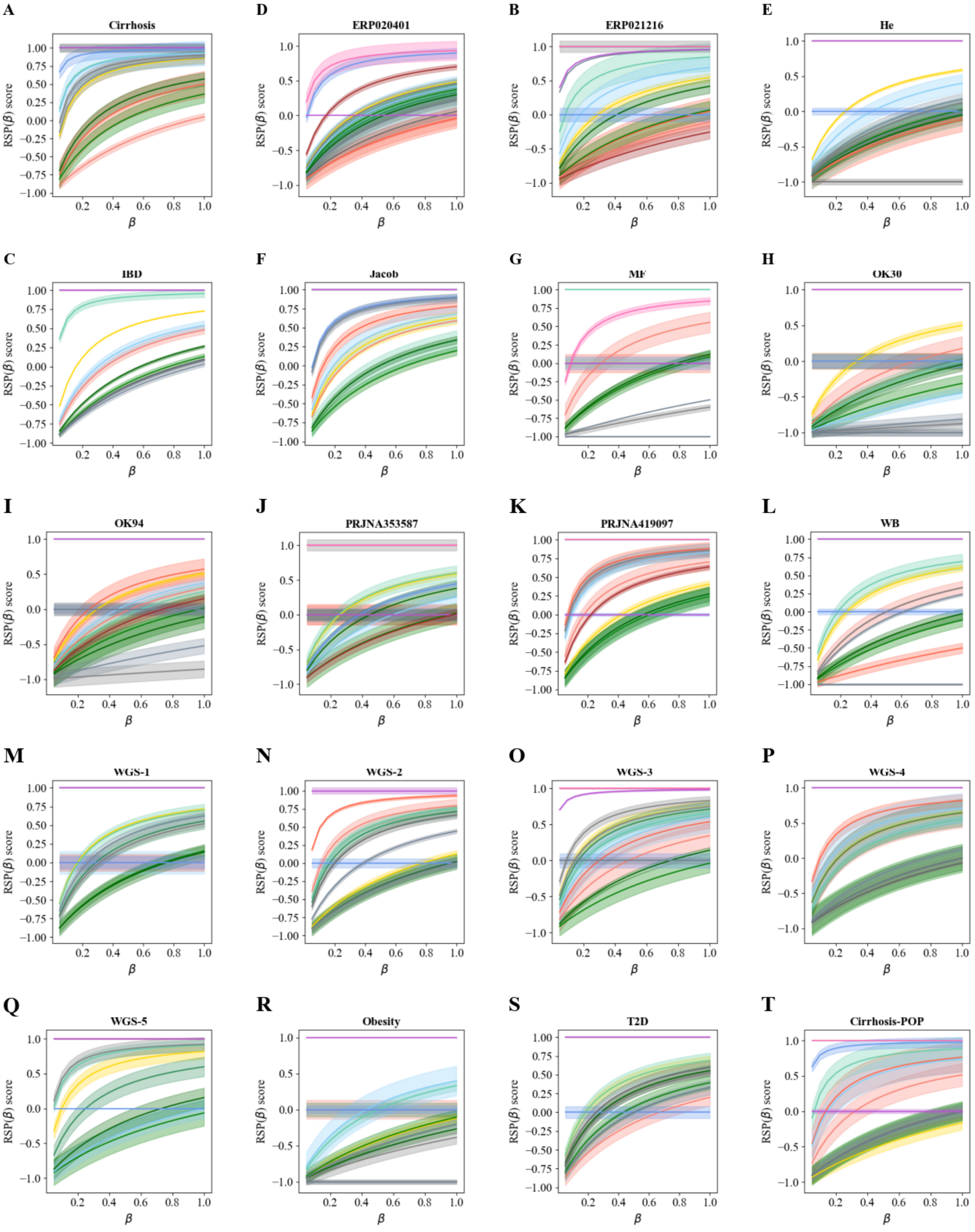
A
Cirrhosis
D
ERP020401
B
ERP021216
E
He
C
IBD
F
Jacob
G
MF
H
OK30
I
OK94
J
PRJNA353587
K
PRJNA419097
L
WB
M
WGS-1
N
WGS-2
O
WGS-3
P
WGS-4
Q
WGS-5
R
Obesity
S
T2D
T
Cirrhosis-POP
RSP(β) score
β

Fig. S2: Comparative analysis of different Differential Analysis (DA) methods as a function of RSP(β) per cohort. Each color represents a model: orange for DeSeq2 (light without FDR correction and dark with FDR correction, denoted as DeSeq2-C), yellow for LefSe, green for ANCOM (light without FDR correction and dark with FDR correction, referred to as ANCOM-C), blue for LINDA (light without FDR correction and dark with FDR correction, denoted as LINDA-C), brown for ada-ANCOM and pink for miMic (pink for log SUB-PCA MIPMLP preprocessing and purple for relative mean MIPMLP preprocessing). Each line illustrates the average RSP(β) score across 10 different shuffles. The light shadows surrounding each line represent standard errors calculated over 10 simulations of the shuffled models.

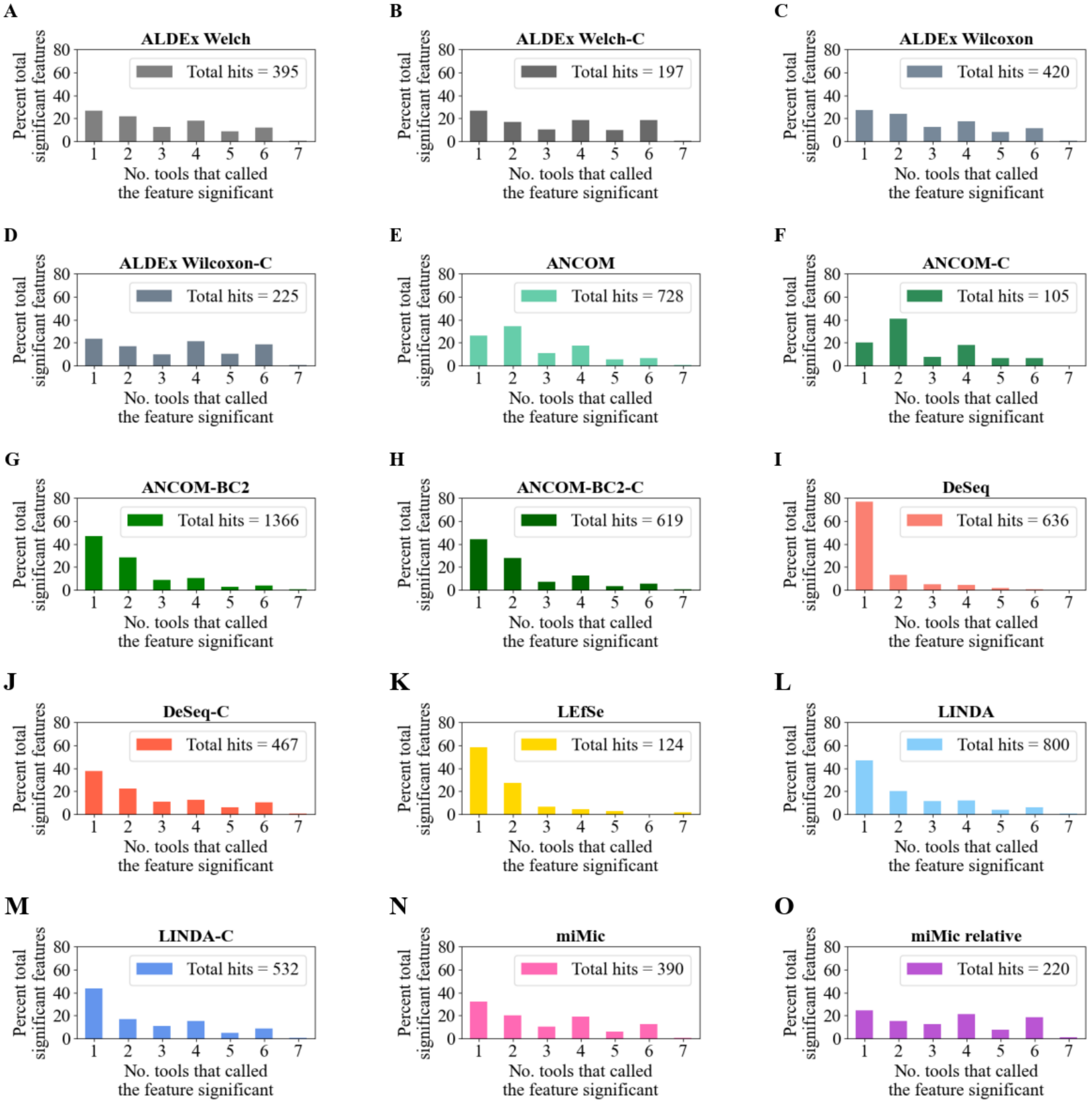

Fig. S3: Within-study differential abundance consistency analysis across multiple tools. The percentage of total significant features is plotted against the number of tools that identified the feature as significant. The total number of significant features identified by each tool is provided in the legend.

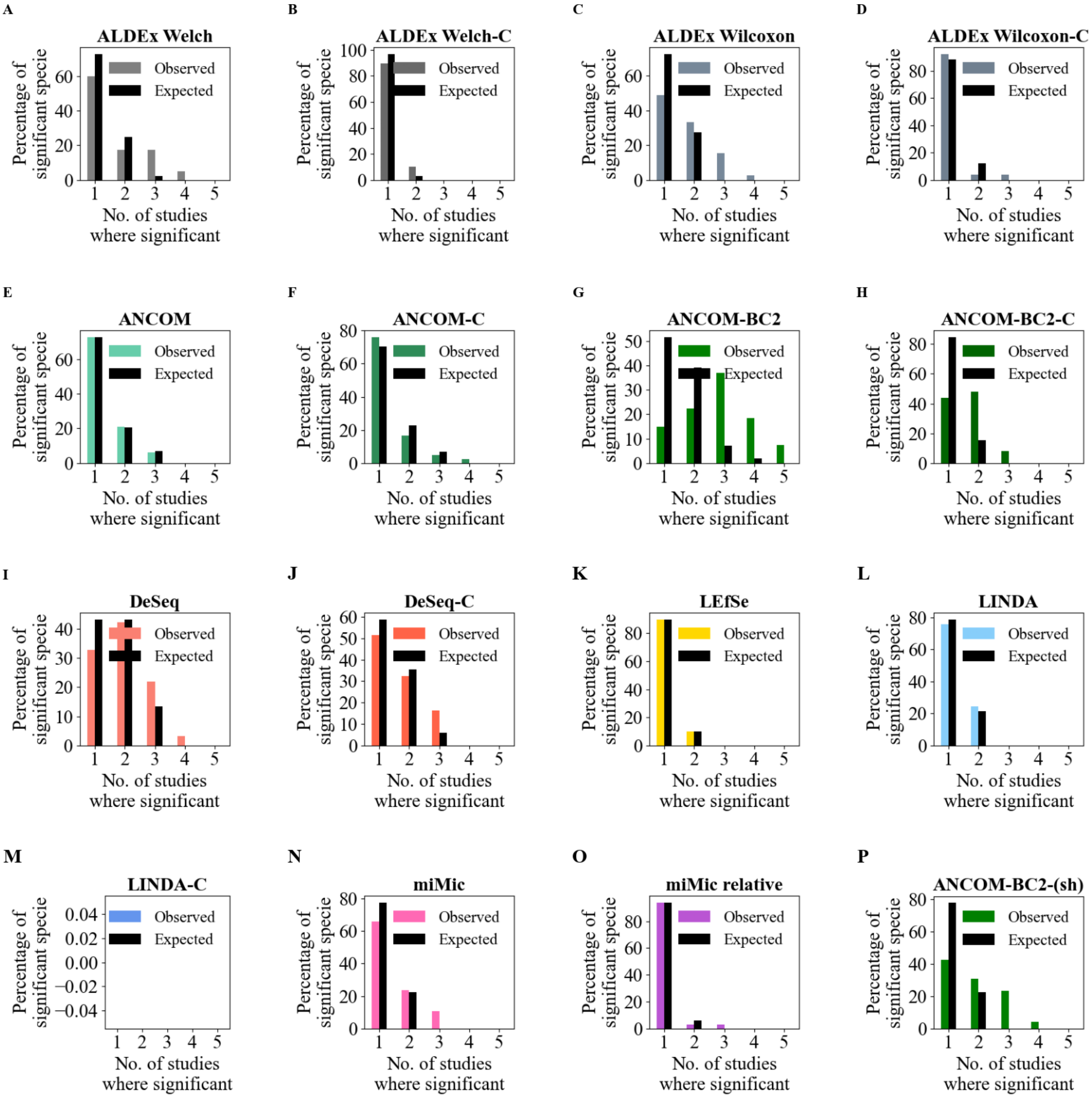

Fig. S4: Cross-study consistency analysis of differential abundance. The percentage of significant species is plotted against the number of studies where each species was identified as significant, conducted on five inflammatory bowel disease (IBD) cohorts. Observed results for each tool are depicted in the tool color, while the expected results are presented in black (see Methods). Additionally, a parallel analysis on shuffled labels is provided for the ANCOM-BC2 model (green) within **(P)**. The models' performance exceeds that of the expected random model. However, certain tools, such as ANCOM-BC2, exhibit artificially consistent results, as indicated in **(P)**. Note that LINDA-C's plot is empty since there were no common significant species between the studies.

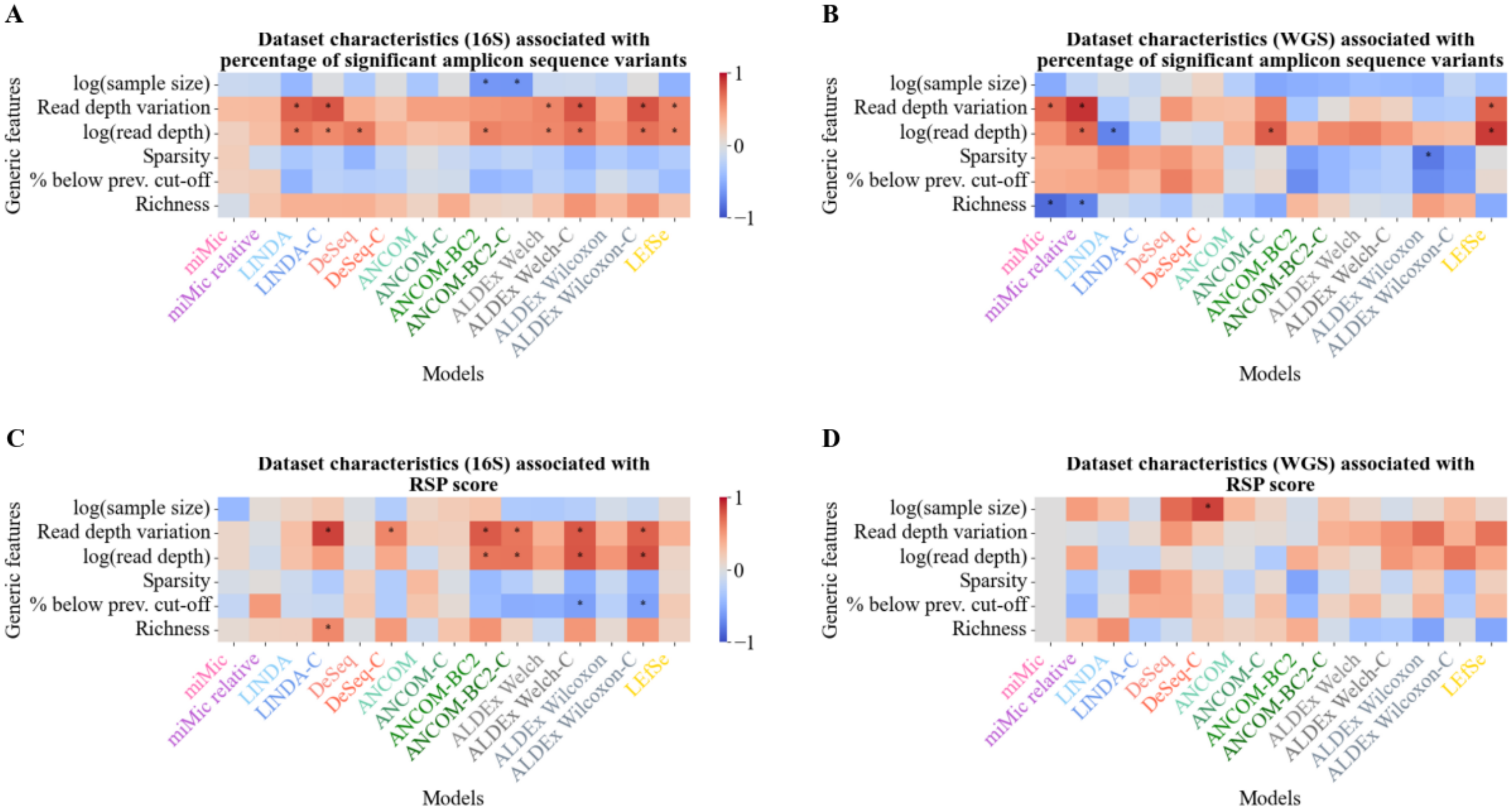

Fig. S5: Sensitivity robustness assessment. **A-B.** The heatmaps illustrate Spearman correlation coefficients (SCCs) between each generic dataset characteristic and the percentage of significant taxa identified by each tool per dataset in 16S **(A)** and WGS **(B)** cohorts. **C-D.** The heatmaps illustrate Spearman correlation coefficients (SCCs) between each generic dataset characteristic and the RSP(1) score per dataset in 16S **(C)** and WGS **(D)** cohorts. Positive correlations are depicted in red, while negative correlations are shown in blue. Stars indicate a significant correlation (p-value < 0.05). miMic demonstrates robustness across all tested generic features in 16S and WGS datasets.

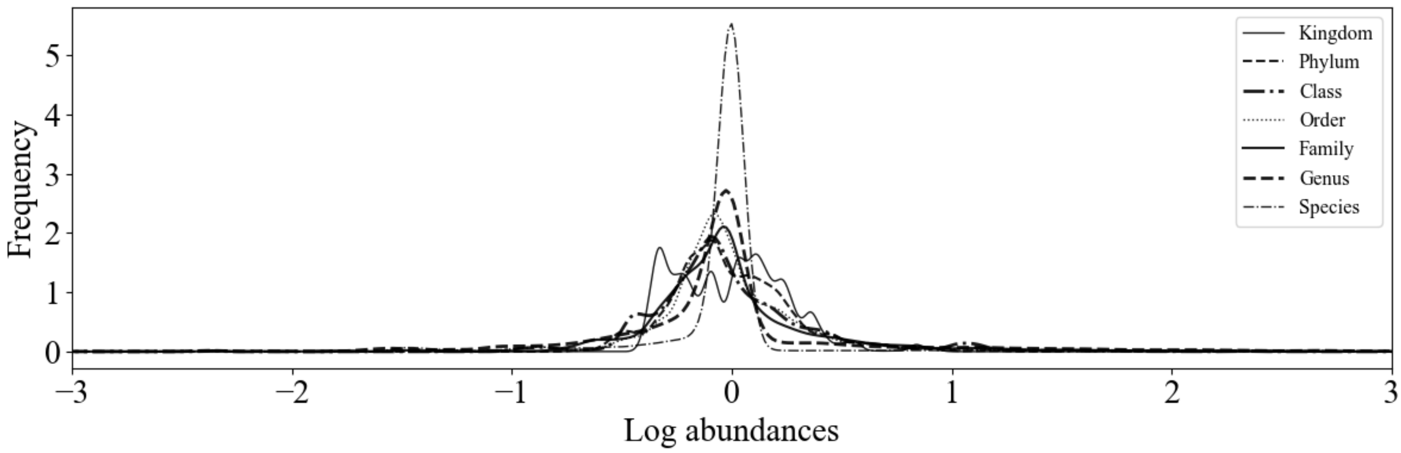

Fig. S6: Histogram of the distribution of logged abundances within each level of taxonomy on the cladogram of means. Different line styles and line weights are assigned to each taxonomy level for distinction. This histogram is based on the IBD cohort.

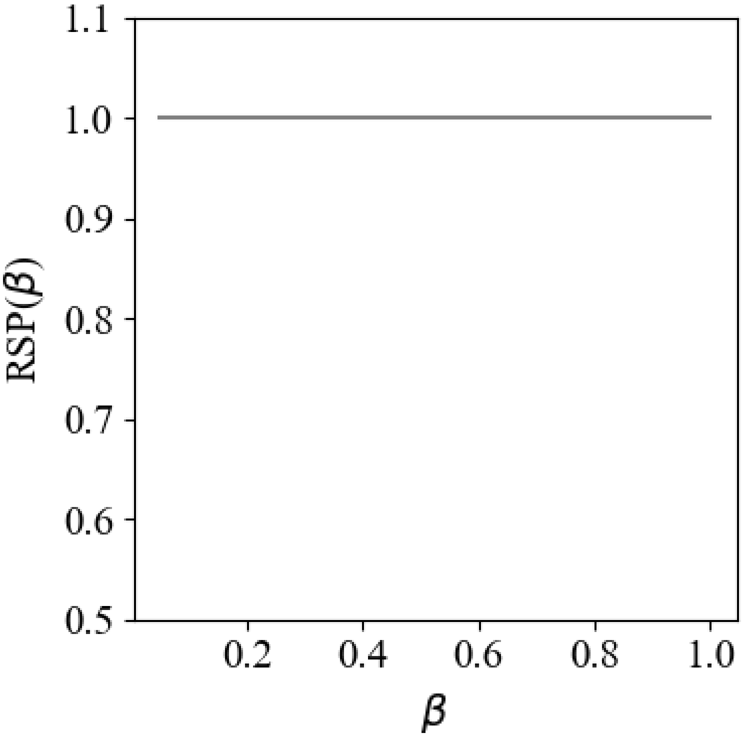

Fig. S7: Representation of RSP(β) score as a function of the confidence level beta on the IBD cohort. An RSP score of 1 indicates the presence of only RP without any SP.

4 Supplementary Tables

Table S1: Acronym Table

**Acronym**	**Meaning**
miMic	Mann-Whitney iMage Microbiome
ANOVA	ANalysis Of VAriance
DNA	Deoxyribonucleic Acid
RNA	Ribonucleic Acid
BMI	Body Mass Index
DTM	Dirichlet-Tree Multinomial
FDR	False Discovery Rate
OLS	Ordinary Least Squares
WGS	Whole Genome Sequencing
OTU	Operational Taxonomic Units
ASV	Amplicon Sequence Variant
LDA	Linear Discriminant Analysis
LEfSe	Linear discriminant analysis Effect SizE
ANCOM	ANalysis of Compositions Of Microbiomes
DeSeq2	Differential analysis of count data
LINDA	LINear models for Differential Abundance analysis
CLR	Centered Log Ratio
GT	Ground Truth
FP	False Positives
FN	False Negatives
TP	True Positives
RP	Real Positives
SP	Shuffled Positives
RSP	Real Positives vs. Shuffled Positives
GLM	Generalized Linear Model
DOF	Degrees Of Freedom
SOTA	State-Of-The-Art
AUC	Area Under the Sensitivity-Specficity Curve
iMic	iMage Microbiome
SCC	Spearman correlation coefficient
IBD	Inflammatory Bowel Disease

Table S2: Datasets details

Dataset name	16S/WGS	Samples	Condition	Origin	Reference
Cirrhosis	16S	130	Cirrhosis	Stool	[1]
Cirrhosis-pop	WGS	232	Cirrhosis	Stool	[2]
ERAWIJANTARI (WGS-1)	WGS	96	Gastrectomy gastric cancer	Stool	[3]
ERP020401 (IBD1)	16S	684	IBD	Stool	[4]
ERP021216 (IBD2)	16S	86	IBD	Stool	[5]
FRANZOSA (WGS-3)	WGS	220	IBD	Stool	[6]
GGMP	16S	7008	Many conditions	Stool	[7]
HE	16S	277	Breastfeeding vs. formula	Stool	[8]
IBD	16S	257	IBD	Stool	[9]
JACOB	16S	90	IBD	Stool	[10]
MF	16S	190	Male vs. female	Stool	[1]
MARS (WGS-5)	WGS	444	IBS	Stool	[11]
Obesity	WGS	253	Obesity	Stool	[2]
OK94	16S	132	CD vs. UC	Stool	[9]
PRJNA353587 (CDI1)	16S	83	CDI	Stool	[12]
PRJNA412501 (CDI2)	16S	71	CDI	Stool	[13]
PRJNA419097 (CDI3)	16S	104	CDI	Stool	[14]
T2D	WGS	342	Diabetes-2	Stool	[2]
WANG (WGS-4)	WGS	287	End-stage renal disease (ESRD)	Stool	[15]
WB	16S	200	White vs. black	Vagina	[16]
YACHIDA (WGS-2)	WGS	347	CRC	Stool	[17]

Table S3: P-values of statistical comparison between models and datasets by two-way ANOVA followed by a one-sided t-test between the two best models.

β	ANOVA dataset	ANOVA model	One-sided t-test between 2 best models
0.05	1.00579524871155e-13	0.0714646978400130	0.0298861393623263
0.1	3.80181980166135e-13	0.0406985427809475	0.00683122299031286
0.5	5.43495185345267e-05	4.86077198185387e-09	0.0134883135956988
1	7.09860323724685e-06	4.80131221816908e-08	0.0107775091701359

Table S4: Different preprocessing of different tools

Tool	Input	Normalization or transformation
LEfSe	Counts	Total sum scaling (TSS)
ANCOM	Counts	Additive log ratio (ALR) transformation
ANCOM-BC2	Counts	1. Log normalization<br>2. TSS<br>3. Pseudocount approach to handle zero values in the data
DeSeq2	Counts	Modified relative log expression (RLE)
ALDEx2	Counts	Centered log ratio (CLR) normalization
LINDA	Relative abundances	Centered log ratio (CLR) normalization
structSSI	1. OTU abundance table<br>2. Phylogenetic tree of OTU/ASV sequences<br>3. Sample grouping information<br>4. Taxonomic information about OTUs to annotate clusters	Total sum scaling (TSS)
PhAAT	1. OTU abundance table<br>2. Phylogenetic tree of OTU/ASV sequences<br>3. Sample grouping information<br>4. Taxonomic information about OTUs to annotate clusters	Relative abundances normalization
ada-ANCOM	1. OTU abundance table<br>2. Phylogenetic tree of OTU/ASV sequences<br>3. Sample grouping information<br>4. Taxonomic information about OTUs to annotate clusters	Posterior mean transformation
miMic relative	Counts	1. Applying MIPMLP including merging ASVs of the same taxonomy by summing, and applying relative normalization.<br>2. Building a cladogram of means.
miMic	Counts	1. Applying MIPMLP including merging ASVs of the same taxonomy by sub-PCA, and applying log normalization.<br>2. Building a cladogram of means.

References

[1] Human Microbiome Project Consortium et al. Structure, function and diversity of the healthy human microbiome. *Nature*, 486(7402):207, 2012.

[2] Derek Reiman, Ahmed A Metwally, Jun Sun, and Yang Dai. Popphy-cnn: a phylogenetic tree embedded architecture for convolutional neural networks to predict host phenotype from metagenomic data. *IEEE Journal of Biomedical and Health Informatics*, 24(10):2993–3001, 2020.

[3] Pande Putu Erawijantari, Sayaka Mizutani, Hirotsugu Shiroma, Satoshi Shiba, Takeshi Nakajima, Taku Sakamoto, Yutaka Saito, Shinji Fukuda, Shinichi Yachida, and Takuji Yamada. Influence of gastrectomy for gastric cancer treatment on faecal microbiome and metabolome profiles. *Gut*, 69(8):1404–1415, 2020.

[4] University of California San Diego Microbiome Initiative. Dynamics of the gut microbiome in inflammatory bowel disease. https://www.ncbi.nlm.nih.gov/sra/?term=ERP020401, 2016.

[5] Sahil Khanna, Yoshiki Vazquez-Baeza, Antonio González, Sophie Weiss, Bradley Schmidt, David A Muñiz-Pedrogo, John F Rainey, Patricia Kammer, Heidi Nelson, Michael Sadowsky, et al. Changes in microbial ecology after fecal microbiota transplantation for recurrent c. difficile infection affected by underlying inflammatory bowel disease. *Microbiome*, 5(1):1–8, 2017.

[6] Eric A Franzosa, Alexandra Sirota-Madi, Julian Avila-Pacheco, Nadine Fornelos, Henry J Haiser, Stefan Reinker, Tommi Vatanen, A Brantley Hall, Himel Mallick, Lauren J McIver, et al. Gut microbiome structure and metabolic activity in inflammatory bowel disease. *Nature Microbiology*, 4(2):293–305, 2019.

[7] Yan He, Wei Wu, Hui-Min Zheng, Pan Li, Daniel McDonald, Hua-Fang Sheng, Mu-Xuan Chen, Zi-Hui Chen, Gui-Yuan Ji, Zhong-Dai-Xi Zheng, et al. Regional variation limits applications of healthy gut microbiome reference ranges and disease models. *Nature Medicine*, 24(10):1532–1535, 2018.

[8] Xuan He, Mariana Parenti, Tove Grip, Bo Lönnerdal, Niklas Timby, Magnus Domellöf, Olle Hernell, and Carolyn M Slupsky. Fecal microbiome and metabolome of infants fed bovine mfgm supplemented formula or standard formula with breast-fed infants as reference: a randomized controlled trial. *Scientific Reports*, 9(1):11589, 2019.

[9] Janine van der Giessen, Dana Binyamin, Anna Belogolovski, Sigal Frishman, Kinneret Tenenbaum-Gavish, Eran Hadar, Yoram Louzoun, Maikel Petrus Peppelenbosch, Christien Janneke van der Woude, Omry Koren, et al. Modulation of cytokine patterns and microbiome during pregnancy in ibd. *Gut*, 69(3):473–486, 2020.

[10] Jonathan P Jacobs, Maryam Goudarzi, Namita Singh, Maomeng Tong, Ian H McHardy, Paul Ruegger, Miro Asadourian, Bo-Hyun Moon, Allyson Ayson, James Borneman, et al. A disease-associated microbial and metabolomics state in relatives of pediatric inflammatory bowel disease patients. *Cellular and Molecular Gastroenterology and Hepatology*, 2(6):750–766, 2016.

[11] Ruben AT Mars, Yi Yang, Tonya Ward, Mo Houtti, Sambhawa Priya, Heather R Lekatz, Xiaojia Tang, Zhifu Sun, Krishna R Kalari, Tal Korem, et al. Longitudinal multi-omics reveals subset-specific mechanisms underlying irritable bowel syndrome. *Cell*, 182(6):1460–1473, 2020.

[12] Tao Zuo, Sunny H Wong, Kelvin Lam, Rashid Lui, Kitty Cheung, Whitney Tang, Jessica YL Ching, Paul KS Chan, Martin CW Chan, Justin CY Wu, et al. Bacteriophage transfer during faecal microbiota transplantation in clostridium difficile infection is associated with treatment outcome. *Gut*, 67(4):634–643, 2018.

[13] University of Colorado School of Medicine. Fecal microbiome among donors and recipients of fecal microbiota transplants. https://www.ncbi.nlm.nih.gov/sra/?term=PRJNA412501, 2017.

[14] The Chinese University of Hong Kong. Bacterial alterions in c.difficile infection and alerations after fecal microbiota transplantation. https://www.ncbi.nlm.nih.gov/sra/?term=PRJNA419097, 2017.

[15] Xifan Wang, Songtao Yang, Shenghui Li, Liang Zhao, Yanling Hao, Junjie Qin, Lian Zhang, Chengying Zhang, Weijing Bian, LI Zuo, et al. Aberrant gut microbiota alters host metabolome and impacts renal failure in humans and rodents. *Gut*, 69(12):2131–2142, 2020.

[16] Jacques Ravel, Pawel Gajer, Zaid Abdo, G Maria Schneider, Sara SK Koenig, Stacey L McCulle, Shara Karlebach, Reshma Gorle, Jennifer Russell, Carol O Tacket, et al. Vaginal microbiome of reproductive-age women. *Proceedings of the National Academy of Sciences*, 108(supplement_1):4680–4687, 2011.

[17] Shinichi Yachida, Sayaka Mizutani, Hirotsugu Shiroma, Satoshi Shiba, Takeshi Nakajima, Taku Sakamoto, Hikaru Watanabe, Keigo Masuda, Yuichiro Nishimoto, Masaru Kubo, et al. Metagenomic and metabolomic analyses reveal distinct stage-specific phenotypes of the gut microbiota in colorectal cancer. *Nature Medicine*, 25(6):968–976, 2019.
