# Supplementary material for: mi-Mic: a novel multi-layer statistical test for microbiota-disease associations
Source: Genome Biol. 2024 May 1;25:113. doi: 10.1186/s13059-024-03256-0 (PMC11064322; doi:10.1186/s13059-024-03256-0)
Supplement: Supplementary file 2 — Additional file 2. Contains the review history. [file 13059_2024_3256_MOESM2_ESM.docx]

**1^st^ round**

**Reviewer 1**

This manuscript presents a new statistical test (miMic) for comparing microbiome profiles between conditions. The tests deals with essential constraints of this type of data: sparsity, compositionality, high dimensionality, abundance variation, and phylogenetic relations and abundance covariation between taxa. The covariance structure is used to mitigate the effect of high dimensionality and multiple testing. The test is scalable and combines earlier statistical techniques in a new way, including data transformation, nested ANOVA, permutation testing, a newly designed post-hoc test. The authors show performance improvements compared to alternative in terms FPR, with the same accuracy. The method is potentially of broad interest to microbiome bioinformatics community but benchmarking with alternatives has limitations as detailed below.

Reporting and language are readable but could be improved by reducing the use of acronyms and aiming at more intuitive descriptions; see major comments for feedback on the figures. The derivations seem OK although I could not check all derivations and proofs in detail.

The choices and conclusions are justified; appropriate references to earlier literature have been provided, with some reservations (see below).


Major:

1) ALDEx2 and ANCOM-BC have performed particularly well in systematic benchmarks (see e.g. Nearing et al. 2022 https://doi.org/10.1038/s41467-022-28034-z but also other recent comparisons). These methods are frequently used in the literature as well, and it is as essential and fair to include comparisons to these methods that been reported to outperform the alternatives than just those methods that are currently highly cited (which is mentioned as the criterion for the present draft manuscript: LEfSe, ANCOM, DeSeq2, LinDA). Hence, the current manuscript does not adequately support the conclusions about the general performance of this method and will limit its general utility (see also comment (4) regarding this).

2) The proposed method is a heuristic combination of established statistical techniques and has certain novelty and interest. For instance, the phylogeny-aware methodology is an interesting contribution as many currently available DA tests do not consider this. More references to earlier related tests could be provided, and where possible, also comparisons (or justification why comparison is not possible). Such tests include: StructSSI (https://pubmed.ncbi.nlm.nih.gov/26917999/) and PhAAT (https://github.com/mruehlemann/phaat) at least.

3) The proposed method has multiple steps, and a more intuitive description could be warranted than what is now provided in Figure 1

4) The method comparisons are based on RSP(beta) score; the benchmarking tests in the literature (e.g. Nearing et al. 2022; see comment (1) and also Weiss et al. 2017 https://doi.org/10.1186/s40168-017-0237-y ) use also other metrics. It would be informative to demonstrate performance using multiple commonly used benchmarking metrics, in order to also understand the possible limitations of miMic better. Under which conditions this method would underperform the alternatives?

5) Figure quality could be generally improved, the figures could be more self-explanatory and be splitted into multiple smaller figures for a better readability; Figure 1A: method performance comparisons would be more essential than the popularity of some given methods; I would suggest removing this figure as non-informative for the contents of this mansucript; 1B: SP vs. RP not explained and color codes should be shown in the figure; 1C: Y axis unit missing and font sizes in the figure unreadable small; 1D: this figure might work better in supplements; Figure 3 is this green-red colorblind accessible? Why Fig3A has question marks? Fig 4 font sizes could be increased; not clear how "code availability" (mentioned in caption title) is illustrated in this figure.

6) An open source implementation is provided with examples. I did not find the source code for the analyses and figures shown in this manuscript, however.

Minor:

- the text refers to "inner structure" and "inner relation" between
microbial taxa; does this refer to covariance between microbial
abundances, the phylogenetic relation, or both? I suggest to use the
established terms for more clarity.

- "microbiota" refers to taxonomic composition and could be better
justified for this manuscript than "microbiome" (see also Marchesi
et al. 2015 for suggested vocabulary for microbiome research)

- Paper mentions ANCOM and ANCOM-C (l. 243/53); does the latter refer
to ANCOM-BC?

- apriori -> a priori

- Appendix, section 6 is empty?

- Project website listed in the manuscript
(https://micros.math.biu.ac.il/) does not open

**Reviewer 2**

The article introduces miMic, a novel approach for microbiome differential abundance analysis. Designed to tackle issues like sparse data, varying abundance scales, and taxonomic relationships, miMic operates through a structured process involving data preprocessing, apriori nested ANOVA, and a post hoc miMic test along cladogram trajectories. It aims to reduce false positives while maintaining high discovery rates. The validation includes analytical models, simulations, and comparisons with existing methods over diverse datasets, demonstrating miMic's accuracy and advantages. However, However, the definitive reason for considering the method a clear winner isn't evident to me. Here are my major comments:

1. The article underscores the difficulty of assessing differential analysis methods without a definitive ground truth, presenting a challenge in conclusively validating the proposed method's accuracy. It is noteworthy that the simulations are crafted to mirror the hierarchical structure of the proposed model, imparting a constructed advantage to the method in question. However, it is acknowledged that these simulations may not fully capture the characteristics of real microbiome data. To enhance the evaluation, the authors are encouraged to explore alternative simulations, such as utilizing the simulation function to mimic real data in the GUniFrac package in R.

2. While miMic is presented as versatile, the complexity of the method, involving multiple steps like data preprocessing, cladogram translation, apriori nested ANOVA, and post hoc miMic test, might be a drawback for users seeking simpler approaches. Specifically, the proposed method necessitates specific preprocessing steps, such as merging similar features based on taxonomy, scaling the distribution, standardizing to z-scores, and dimension reduction. Concerns are raised regarding the potential loss of candidates during the feature merging and dimension reduction stages. An important question arises: what would be the outcome if the preprocessing steps are omitted, and miMic is applied directly to the data? Additionally, it would be valuable to know whether preprocessing was applied to the original data before subjecting it to other state-of-the-art methods.

3. The validation of miMic is based on analytical models, simulations, and real- world cases. However, the article should discuss potential limitations or cases where miMic may not perform optimally. The method's boundaries and capabilities have not been thoroughly elucidated.

4. The article notes that miMic obtains the best results when starting on specific taxonomy levels. This sensitivity to the cladogram structure may limit its generalizability to diverse datasets.

5. While the article emphasizes the importance of considering the correlation structure among taxa, it would be useful to discuss how miMic handles cases where taxa have complex and nonlinear relationships.

6. The challenge of differential analysis varies between the majority and minority microbiome. It appears that the proposed method excels in detecting abundant features compared to scarce features. Because the paper mentioned it exhibits more power in identifying upper-level nodes than lower-level nodes. Is this true? Are there any validations?

7. The theoretical calculations are somewhat perplexing. In Equation (3) of section 7.1, m-significant is denoted, while in Equation (10) of section 7.2, f- significant is mentioned. It's unclear what f represents in this context. Moreover, the assertion that from the joint distribution, one can conclude with high probability that if sisters are not associated with the class, neither is their mother, seems non-trivial. Please elaborate it in more details.

8. In the captions of Figure 4, plot E is not described. The current descriptions for plot E are meant for plot F. Please check the captions and descriptions of all the other figures

**Authors’ response**

Reviewer 1

This manuscript presents a new statistical test (miMic) for comparing microbiome profiles between conditions. The tests deals with essential constraints of this type of data: sparsity, compositionality, high dimensionality, abundance variation, and phylogenetic relations and abundance covariation between taxa. The covariance structure is used to mitigate the effect of high dimensionality and multiple testing. The test is scalable and combines earlier statistical techniques in a new way, including data transformation, nested ANOVA, permutation testing, a newly designed post-hoc test. The authors show performance improvements compared to alternative in terms FPR, with the same accuracy. The method is potentially of broad interest to microbiome bioinformatics community but benchmarking with alternatives has limitations as detailed below.

Reporting and language are readable but could be improved by reducing the use of acronyms and aiming at more intuitive descriptions; see major comments for feedback on the figures. The derivations seem OK although I could not check all derivations and proofs in detail.

The choices and conclusions are justified; appropriate references to earlier literature have been provided, with some reservations (see below).

R1. We sincerely appreciate your thorough review of our manuscript and are grateful for your approval of miMic. We have carefully considered and addressed the limitations in benchmarking that you highlighted during the major revisions. In response to your comments, we have expanded our comparisons to include more state-of-the-art models (ANCOM-BC2, ALDEx2, structSSI, and ada-AMCOM were added), aiming for a more comprehensive evaluation. Additionally, we have incorporated additional evaluation methods, such as consistency and sensitivity tests, alongside with the new proposed RSP measure, to provide a more holistic assessment of miMic's performance. We have also enlarged our references to earlier literature. We have rewritten sections of the manuscript to ensure clarity and accessibility to a wide audience, as per your suggestions. We have also added a detailed acronym table to ease the reading of acronyms (Table S1).

Table S1: Acronym table

Major:

1) ALDEx2 and ANCOM-BC have performed particularly well in systematic benchmarks (see e.g. Nearing et al. 2022 https://doi.org/10.1038/s41467-022-28034-z but also other recent comparisons). These methods are frequently used in the literature as well, and it is as essential and fair to include comparisons to these methods that been reported to outperform the alternatives than just those methods that are currently highly cited (which is mentioned as the criterion for the present draft manuscript: LEfSe, ANCOM, DeSeq2, LinDA). Hence, the current manuscript does not adequately support the conclusions about the general performance of this method and will limit its general utility (see also comment (4) regarding this).

We sincerely appreciate the neat Nearing et al. [1] paper that we have now cited in our manuscript. Building upon this valuable input, we have applied ALDEx2 and ANCOM- BC2 to all datasets analyzed in the manuscript (n = 20), both with and without multiple test corrections, utilizing their various statistical test options when available.

Consequently, we have expanded our comparisons to include these two prominent methods in all evaluations, as outlined in our response to comment 4 in the revised manuscript (Fig. 1 B, Fig. 3, Supp. Mat. Fig. S2). miMic is more accurate than these state-of-the-art models, exhibiting stable real positives (RP) and low shuffled positives (SP). We have also added consistency tests within different cohorts with the same phenotype (for more details see our response in 4 and Fig. 4 and Supp. Mat. Fig. S3- S4), consistency of significant taxa found within different methods (for more details see our response in 4) and sensitivity tests to generic technical features of the cohort (such as read depth, sparsity, richness, cohort’s size, etc.), further affirming miMic's robustness and reliability across diverse scenarios (Fig. 4 D and Supp. Mat. Fig. S5).

These texts were added to the revised manuscript: “ANCOM-BC2 extends ANCOM by incorporating additional blocking and covariate terms into the model through multivariate regression analysis. DeSeq2 models variance-mean dependence in count data using the negative binomial distribution. ALDEx2 employs Monte Carlo sampling and Bayesian modeling to estimate technical variability and sampling uncertainty in compositional microbiome data. LINDA performs a linear regression with CLR (centered log ratio)-transformed abundance data, corrects for compositional bias, calculates p-values based on bias-adjusted coefficients, and controls the false discovery using the Benjamini-Hochberg procedure. “(Lines 40-47). “We next investigated the overlap in significant taxa across tools within each dataset [1]. While this is not an absolute measure of accuracy, consistency implies that the assumptions made in the analysis have a limited effect on the results. If two methods with different assumptions produce consistent results, one can assume that the results are not strongly affected by the assumptions. miMic is more consistent with other models than the average of all models (Fig. 4 A and Supp. Mat. Fig. S3 - where miMics' distribution of overlap is more skewed to higher overlap values than the average overlap distribution of the other models). We next investigated the consistency of miMic (vs. other models) across datasets of the same disease. We specifically focused on IBD as a phenotype, which has been shown to exhibit a strong effect on the microbiome and to be relatively reproducible across studies [11,12]. We acquired five datasets for this analysis representing the microbiome of individuals with IBD compared with individuals without IBD (see Methods). We ran all differential abundance analysis tools on each individual dataset and restricted our analyses to the 81 species found across all datasets. Tools that generally identify more species as significant are accordingly more likely to identify species as consistently significant compared with tools with fewer significant hits. We thus compared the observed distribution against the expected distribution given random labels. miMic demonstrates notable consistency in findings across different cohorts, with over 10% of significant species consistent within three different cohorts (Fig. 4 B). While other models show similar behavior on average, miMic stands out for its lack of false consistent species. Notably, all tools exhibit significantly higher consistency than random expectation across these datasets (Fig. 4 B and C and Supp. Mat. Fig. S4). Another limitation of a statistical tool may be the effect of technical aspects of the sample on the fraction of real-label positives and shuffled positives in different datasets. We analyzed the correlation between multiple characteristics such as read depth, sparsity, richness, and dataset size and the percentage of significant taxa identified by each tool per dataset. In contrast with most other methods, we found no significant correlations of any of these factors with the miMics' percentage of significant taxa (Fig. 4 D). However, as expected, we do find in WGS correlations of the richness with the number of positive results (since there are more species tested - Supp. Mat. Fig. S5).” (Lines 289-316).

Figure 1: Challenges in Difference Analysis of Microbiome. A. Pie diagram illustrating the usage of differential analysis methods within the field during 2023. The colors represent different methods: LefSe (yellow), DeSeq2 (orange), ANCOM (sea-green), ANCOM-BC2 (green), ALDEx2 (grey), and LINDA (light blue). B. Scatter plot comparing the performance (SP vs. RP) of popular methods across more than 20 different microbial datasets. Each shape represents a distinct cohort with colors matching those indicated in the pie chart A. Dark colors indicate methods with FDR corrections, while light colors represent methods without any corrections. The dashed grey line represents the y = x line, where the SP rate is similar to the RP rate. Pink and purple colors in the upper left corner represent miMic and miMic-relative, respectively. In many cases (excluding miMic), the RP and SP rates are similar. C. Inner sisters'- labels SCCs over the different datasets. The stars represent the significance of the correlations, such that -p<0.05, **-p<0.01, ***-p<0.001. D. Schematic explanation of the miMic approach. miMic is a three-step method: (1) Data processing (in light green). Microbiome data preprocessing involves using the MIPMLP method (see Methods) and converting it into a cladogram of means using the iMic algorithm (see Methods). (2) A priori nested ANOVA (in light blue). A priori nested ANOVA (nested GLM for continuous labels) is applied to microbiome cladograms and labels to assess the global relationship between labels and the entire microbiome. If no significant relationship is found at any taxonomy level, the label is defined "not microbially explainable," and no further analysis is performed. (3) Post hoc Mann-Whitney test. A post hoc Mann- Whitney test, leveraging phylogenetic information, is conducted for the difference analysis (1). Starting at the first taxonomy level, a Mann-Whitney (or Spearman correlation) test is applied to each taxon's values, with a predetermined significance level (currently p < 0.05). Multiple measurement correction is applied only for the number of taxa at this level. If significant taxa are identified, the analysis iteratively proceeds along the cladogram towards the leaf nodes. To address inner sister relations, FDR control is used when multiple significant sisters are present. An additional Mann-Whitney test (utilizing Spearman correlation for continuous labels) is specifically conducted over the leaves, incorporating FDR correction for multiple measurements (2). miMic consolidates the significant taxa identified in steps 1 and 2.

The schematic cladogram in the bottom right corner illustrates the inner taxa of the cladogram utilized in step 1 of the post hoc test (grey), while its white leaves are employed in both steps. "+/-" denotes samples with/without the phenotype, respectively. A white circle marked "p" represents a p-value. An orange circle labeled "Sis" signifies FDR correction applied over the sisters, as explained in the manuscript. A green square or circle labeled "s" indicates a significant taxon, while a red square or circle marked "n.s" indicates an insignificant taxon.

Figure 3: Validation of miMic vs. SOTA models on real-world datasets. A. RSP(β) schematic explanation. The tasks in the differential analysis field can be divided into 2 main types: (1) tasks with a predefined GT, where False and True labels are clearly defined (upper left scheme), and (2) tasks without a predefined GT, as is common in most real-world datasets (upper right scheme). In the second scenario, "True" and "False" are replaced with "Real" (calculated on real labels) and "Shuffled" (calculated on shuffled labels). The RSP(β) score is defined by (β *RP -SP)/ (β *RP+SP)), where β representing the user-defined weighting of Real Positives (RP) vs. Shuffled Positives (SP). B - C. Comparative analysis of different differential analysis methods as a function of RSP(β) over 16S cohorts (B) and WGS cohorts (C). Each color represents a specific model: orange for DeSeq2 (light without FDR correction and dark with FDR correction, denoted as DeSeq2-C), yellow for LefSe, green for ANCOM (light without FDR correction and dark with FDR correction, referred to as ANCOM-C), blue for LINDA (light without FDR correction and dark with FDR correction, denoted as LINDA- C), brown for ada-ANCOM, and pink for miMic (pink for log Sub-PCA MIPMLP preprocessing and purple for relative mean MIPMLP preprocessing). Each line illustrates the average RSP(β) score across all cohorts (12 16S in B and 8 WGS in C). The light shadows surrounding each line represent standard errors calculated over 10 simulations of the shuffled models across all cohorts (12 16S in B and 8 WGS in C). D - E. Comparison of different starting taxonomy levels of the miMic test and their corresponding RSP(β) scores over 16S cohorts (D) and WGS cohorts (E). Each taxonomy level is indicated by a different line style (1 for kingdom, 2 for phylum, 3 for class, 4 for order, 5 for family, 6 for genus, and 7 for species). Typically, the best RSP scores are achieved in the first two taxonomy levels. The inner bar plot presents the number of cohorts in which miMic is deemed significant when commencing the Mann- Whitney test at each taxonomy level. F. Scatter plot of the sister's-labels SCC vs. the RSP(1) score. A significant positive correlation of 0.588 is observed between the SCCs of sister labels and the model's performance.

Figure S2: Comparative analysis of different Differential Analysis (DA) methods as a function of RSP(β) per cohort. Each color represents a model: orange for DeSeq2 (light without FDR correction and dark with FDR correction, denoted as DeSeq2-C), yellow for LefSe, green for ANCOM (light without FDR correction and dark with FDR correction, referred to as ANCOM-C), blue for LINDA (light without FDR correction and dark with FDR correction, denoted as LINDA-C), brown for ada-ANCOM and pink for miMic (pink for log SUB-PCA MIPMLP preprocessing and purple for relative mean MIPMLP preprocessing). Each line illustrates the average RSP(β) score across 10 different shuffles. The light shadows surrounding each line represent standard errors calculated over 10 simulations of the shuffled models.

Figure S3: Within-study differential abundance consistency analysis across multiple tools. The percentage of total significant features is plotted against the number of tools that identified the feature as significant. The total number of significant features identified by each tool is provided in the legend.

Figure S4: Cross-study consistency analysis of differential abundance. The percentage of significant species is plotted against the number of studies where each species was identified as significant, conducted on five inflammatory bowel disease (IBD) cohorts. Observed results for each tool are depicted in the tool color, while the expected results are presented in black (see Methods). Additionally, a parallel analysis on shuffled labels is provided for the ANCOM-BC2 model (green) within (P). The models' performance exceeds that of the expected random model. However, certain tools, such as ANCOM-BC2, exhibit artificially consistent results, as indicated in (P). Note that LINDA-C's plot is empty since there were no common significant species between the studies.

Figure 4: Consistency and robustness analysis of miMic. A. Within-study differential abundance consistency analysis across multiple tools. The percentage of total significant features is plotted against the number of tools that identified the feature as significant. Results are shown for the miMic model (pink) and the average of all SOTA models (white). Refer to Supp. Mat. Fig. S3 for a detailed analysis of all 13 tools. The total number of significant features identified by each tool is provided in the legend. miMic demonstrates slightly higher consistency compared to the average of all SOTA models. B-C. Cross-study consistency analysis of differential abundance. The percentage of significant species is plotted against the number of studies where each species was identified as significant, conducted on five IBD cohorts. Results for miMic are depicted in pink (B), while those for the average SOTA model are shown in white (C). The expected results are presented in black (see Methods). Additionally, a parallel analysis on shuffled labels is provided for the ANCOM-BC2 model (green) within (C). The models' performance exceeds that of the expected random model. However, certain tools, such as ANCOM-BC2, exhibit artificially consistent results, as indicated in the inner plot of (C). For a comprehensive analysis of all 13 tools, refer to Supplementary Material Fig. S4. D. Sensitivity robustness assessment. The heatmap illustrates the SCCs between each generic dataset characteristic and the percentage of significant taxa identified by each tool per dataset. Positive correlations are depicted in red, while negative correlations are shown in blue. Stars indicate a significant correlation (p-value < 0.05). miMic demonstrates robustness across all tested generic features in 16S datasets. For parallel analyses conducted on 16S and WGS cohorts, detailing the percentage of significant taxa identified by each tool per dataset and RSP score, refer to Supplementary Material Fig. S5.

Figure S5: Sensitivity robustness assessment. A-B. The heatmaps illustrate Spearman correlation coefficients (SCCs) between each generic dataset characteristic and the percentage of significant taxa identified by each tool per dataset in 16S (A) and WGS (B) cohorts. C-D. The heatmaps illustrate Spearman correlation coefficients (SCCs) between each generic dataset characteristic and the RSP(1) score per dataset in 16S (C) and WGS (D) cohorts. Positive correlations are depicted in red, while negative correlations are shown in blue. Stars indicate a significant correlation (p-value < 0.05). miMic demonstrates robustness across all tested generic features in 16S and WGS datasets.

2) The proposed method is a heuristic combination of established statistical techniques and has certain novelty and interest. For instance, the phylogeny-aware methodology is an interesting contribution as many currently available DA tests do not consider this. More references to earlier related tests could be provided, and where possible, also comparisons (or justification why comparison is not possible). Such tests include: StructSSI (https://pubmed.ncbi.nlm.nih.gov/26917999/) and PhAAT (https://github.com/mruehlemann/phaat) at least.

Indeed, incorporating phylogenetic structure into statistical tests is not yet commonplace in the field, but the differential abundance analysis suffers from a high false-positive rate problem. We utilize the trajectories of the cladogram to reduce the number of measurements (as further explained in the Introduction of the revised manuscript). To provide a broader context for miMic's approach, we have also added a new paragraph to the revised manuscript to review previous phylogeny-aware methods such as structSSI [2], PhAAT [3], and adaANCOM [4], and compare their underlying logic to that of miMic. This section elucidates the unique contributions and advantages of miMic within the landscape of phylogeny-aware statistical methods. The text that was added to the revised manuscript reads as follows: “Among the less common methods are those that incorporate phylogenetic information into their statistical frameworks. For example, structSSI [2]adopts a hierarchical false discovery rate (FDR) control strategy, organizing hypotheses along a phylogenetic tree to enhance the detection of significant signals. Specifically, children's hypotheses are considered for rejection if and only if their parents are rejected. PhAAT (Phylogeny-aware Abundance Testing), https://github.com/mruehlemann/phaat, constructs a Branch-Abundance matrix by multiplying the sequence abundance table with the binary Sequence-to- Branch matrix, enabling representation of branch abundance. PhAAT then conducts filtering steps to reduce multiple testing burdens, applies statistical tests for differential abundance, refines signals by clustering sub-branches with the same effect direction, and annotates final signals based on taxonomic annotations weighted by abundance. ada-ANCOM [4] extends the Dirichlet-tree multinomial (DTM) to zero-inflated DTM, addressing data sparsity (like ANCOM-2) and incorporating phylogeny. It introduces a Bayesian formulation with posterior mean transformation to convert raw counts into non-zero relative abundances, facilitating adaptive analysis of the composition of microbiomes for differential abundance testing.” (Lines 48-62). In addition, we added the phylogeny-aware methods to the comparison between all SOTA methods and miMic in Table 1.

Table 1: Comparison between existing differential analysis methods considering the 'Normalization' - normalization applied during the method, 'Normality assumption' - whether the method assumes the microbiome input is normally distributed, 'Inner relations' - how the method treats the inner relations between the taxa, and 'Multiple measurements' - how the method treats the problem of multiple measurements.

Additionally, we attempted to apply each of the phylogeny-aware methods to real cohorts for a comparative analysis. However, we encountered challenges with the structSSI method, as it is an older method from 2014 and its package is no longer supported. Despite this limitation, we managed to apply structSSI to the original dataset from its own manuscript, and we applied miMic to the same dataset for a comparative assessment. We calculated the real positives (RP) and shuffled positives (SP) of each model under two significance thresholds: once with the conventional p- value < 0.05 criterion and once with the p-value < 0.1 criterion as defined in the structSSI paper [2]. miMic outperforms structSSI under both significance definitions, as summarized in Table R1.

Table R1: Comparison between structSSI and mimic on the chlamydiae dataset.

RPSPRSP(1)

StructSSI (p=0.1)20140.176

miMic (p=0.1)1201

StructSSI (p=0.05)00-

miMic (p=0.05)701

Regarding PhAAT [3], despite our efforts, we were unable to find a published manuscript for this method. Although the Git repository was published six years ago, the application of PhAAT appears to be multilevel precluding its practical implementation in our analysis. Consequently, we have provided a theoretical discussion of PhAAT in the Introduction of the revised manuscript to acknowledge its existence and potential relevance.

Another notable phylogeny-aware model, ada-ANCOM [4], was applied to all datasets for which a phylogenetic tree in Newick format was available (see above Fig. 3 B and Supp. Mat. Fig. S2). This comparative analysis further underscores miMic's competitive performance and versatility across diverse datasets and analytical contexts.

3) The proposed method has multiple steps, and a more intuitive description could be warranted than what is now provided in Figure 1

Indeed, miMic comprises multiple steps, and enhancing the clarity of its description is essential. To address this concern, we have revised the depiction of miMic's methodology in Figure 1D (see above the revised Fig. 1 D). By simplifying the representation of each step, we aim to offer a clearer and more intuitive understanding of the model's workflow. We believe that these changes will facilitate a better grasp of miMic's intricacies, ensuring that readers can comprehend its methodology with greater ease. We have also clarified the explanation.

4) The method comparisons are based on RSP(beta) score; the benchmarking tests in the literature (e.g. Nearing et al. 2022; see comment (1) and also Weiss et al. 2017 https://doi.org/10.1186/s40168-017-0237-y ) use also other metrics. It would be informative to demonstrate performance using multiple commonly used benchmarking metrics, in order to also understand the possible limitations of miMic better. Under which conditions this method would underperform the alternatives?

We have now extended the evaluation of miMic to incorporate the proposed methods.

Our evaluation methods can be categorized into three main types: quality evaluations, consistency evaluations, and sensitivity evaluations.

Regarding the quality evaluations, the problem with the current evaluation methods is that they separate the evaluation of real positives (the significant ASVs that were found) measured by their number or their percent from the total number of ASVs from the evaluation of false positives measured by the number of false discoveries (number/percent of significant ASVs in a shuffled data), leading to methods that favor one or the other. To address this, we introduced the Real vs. Shuffled Positives (RSP(β)) score, which provides a balanced assessment of miMic's performance. We have further extended the plot illustrating the ratio between real positives (RP) and shuffled positives (SP) across different benchmarking tests, with miMic demonstrating the better ratio in miMic (Distance from diagonal of pink and purple in Fig. 1 B above). In terms of consistency evaluations, we have incorporated two additional tests into our revised manuscript:

Cross-Tool, Within-Study Differential Abundance Consistency Analysis: This analysis compares the consistency between different tools across all datasets, pooling all ASVs identified as significant by at least one tool in the 12 16S datasets (above Fig. 4 A and Supp. Mat. Fig. S3).

Cross-Study Differential Abundance Consistency Analysis: We applied each model to five IBD cohorts and assessed the consistency of significant species across datasets.

We computed empirical distributions to determine the expected mean number of studies where a species would be identified as significant, given random sampling (above Fig. 4 B and C and Supp. Mat. Fig. S4).

We have also introduced a sensitivity test to evaluate how generic cohort features, such as read depth, sparsity, richness, and dataset size, influence miMic's performance. This sensitivity analysis provides valuable insights into miMic's robustness across diverse datasets (above Fig. 4 D and Supp. Mat. Fig. S5). These comprehensive evaluations underscore miMic's advantages over existing tools in terms of performance quality, consistency within models and cohorts, and robustness across datasets with varying characteristics.

The evaluation methods mentioned in Weiss et al. require a ground truth therefore they are appropriate for simulations (as used in the original paper) and not for real datasets. In the revised manuscript, we added a realistic microbiome-based simulation in addition to the hierarchical generic simulation, there we computed the F1 score (which considers both type 1 error and type 2 error) over different scenarios. Once again, our results show that miMic's performance outperforms all existing state-of-the-art solutions (Fig. 2 M).

Regarding miMic's limitations, there are two primary constraints. Firstly, when lower taxonomy levels exhibit opposite relationships with the phenotype, the contributions of these levels may cancel each other out at higher taxonomy levels where values are averaged. Secondly, rare taxa whose contributions to higher levels are minor may not be adequately reflected. To address the latter limitation, we have expanded miMic to include significant leaves identified through the Mann-Whitney test, with multiple measurements correction using Bonferroni adjustment, even if they have no ancestor that is significant. By doing so, we ensure that only rare taxa in intermediate taxonomy levels are not omitted from the analysis, thus mitigating this limitation. Note that as shown in the revised manuscript, this increases the RP rate, and practically does not affect the SP. However, the main limitations of miMic emerge from the inherent analysis of microbiome samples that have a very limited overlap between datasets. Indeed, the fraction of taxa overlapping between any two datasets is typically less than 0.1 [6]. Thus, differential expression analysis in per-definition is often not well transferred between datasets. However, the requirement of consistent associations along the cladogram trajectory ensures that at least at a coarse taxonomical description, such association can be maintained. The limitations are directly discussed in the revised manuscript.

Figure 2: Validation of miMics' assumptions on analytical models and simulations. A. Daughter's distribution (pink) vs. mother's distribution (dark pink) in the regime of (0,0,0). The mother's distribution is noticeably narrower, with approximately half that of the daughter's distribution. B - D. Comparison of the leaf confidence with miMic's confidence based on analytical integral calculations over 3 different regimes: regime (0,0,0) (B), regime (0,0,μ) (C), regime (0,μ,αμ) (D). The lines represent estimated slopes, denoted as S. In D, different colors represent varying levels of connection between the sisters, controlled by α values (0.25, 0.5, 1, 2, 4). E. Histogram illustrating the distribution of inner sisters-label SCCs across different cohorts. The black line represents the zero line, and the dashed pink line represents the average of the distribution, indicating a right-skewed distribution, with most sisters showing a consistent positive correlation with the label. F - G. A comparison between the number of samples and the number of FP for miMic and Mann-Whitney leaf simulations based on the regime of (0, 0, 0) (F) and the regime of (0, 0, μ), where μ was set to 1. The lightest pink color represents the Leaf-C model, the middle pink represents the Leaf model, and the darkest pink represents the miMic model. H. Comparison between the number of samples and the number of TP for miMic and Leaf Mann-Whitney simulations based on the regime of (0, 0, μ). The color coding is similar to the color coding of F -G. I - J. Comparison between the FP (I) and TP (J) of the miMic model and the Leaf model in the simulation of the regime (0, 0, μ) over different numbers of samples and different values of μ.The Leaf model shows a higher number of FP compared to miMic, while miMic's TP taxa are similar to Leaf's TP taxa. K - L. Comparison between the FP (K) and TP taxa (L) of the miMic model and the Leaf model in the simulation of the regime (0, μ, αμ) over different numbers of samples and different values of α. The Leaf model exhibits a higher number of FP compared to miMic, while miMic's TP are similar to the Leaf's TP. M. The F1 scores of different differential abundance methods are depicted across three distinct setups of microbiome-oriented simulations. miMic is again pink and is much higher than all current methods, many of which have an F1 of 0.

5) Figure quality could be generally improved, the figures could be more self- explanatory and be splitted into multiple smaller figures for a better readability; Figure 1A: method performance comparisons would be more essential than the popularity of some given methods; I would suggest removing this figure as non-informative for the contents of this manuscript; 1B: SP vs. RP not explained and color codes should be shown in the figure; 1C: Y axis unit missing and font sizes in the figure unreadable small; 1D: this figure might work better in supplements; Figure 3 is this green-red colorblind accessible? Why Fig3A has question marks? Fig 4 font sizes could be increased; not clear how "code availability" (mentioned in caption title) is illustrated in this figure.

We were trying to have each figure summarizing a claim. As such, we attempted to merge all the elements associated with one claim into a single figure. Also, we agree with the reviewer that the method’s popularity does not necessarily indicate the method’s quality. Therefore, the popularity of each model does not come instead of the quality tests, such as RP vs. SP (above Fig. 1 B), the RSP(β) score (above Fig. 3 B-C and Supp. Mat. Fig. S2), consistency tests (above Fig. 4 A-C and Supp. Mat. Fig. S3- S4), and robustness and generality tests (above Fig. 4 D and Supp. Mat. Fig. S5). However, the usage frequency gives the reader the distribution of usage of the existing methods to understand the relation between usage and accuracy (not really related). We also use this information as color coding for the quality test in B. Regarding Fig. 1B we added the meaning of RP and SP in the axes labels. The color coding is in A as explained above and the different shapes that represent the different datasets are presented in C in grey. Regarding Fig. 1C, the Y-axis unit is SCC (Spearman correlation coefficient) as explained in the text. We enlarged the font sizes and have added the shapes in Fig 1B to ease the understanding. Regarding Fig. 1D, we have simplified the plot according to the reviewer's suggestion. However, we believe this figure is crucial for understanding the flow of miMic, so we have retained it in the main manuscript rather than moving it to the supplementary material. Regarding Fig. 3 A, we have addressed the confusion in Figure 3A by removing the question marks and ensuring that the color scheme is green-red colorblind accessible. We redesigned Fig. 4 in the revised manuscript. It has been separated into two different figures in the revised manuscript, Fig. 5 and Fig. 6. Additionally, two of its subplots were moved into the Supplementary Material for clarity. We hope this revision makes it clearer. We thank the reviewer for his remark that it is not clear how "code availability" (mentioned in the caption title) is illustrated in this figure. In response, we have removed the reference to code availability from the title caption in the revised manuscript. Instead, we provide details about code availability separately in the Supplementary Material, along with comprehensive running examples available on PyPi [7] and the micrOS website [8]

Figure 5: Differential abundance analysis results are visualized on a cladogram, where each color represents the sign of the Mann-Whitney score (blue for positive scores, red for negative scores, and grey for non-significant taxa). The node size corresponds to - log10(p-value) from the Mann-Whitney test in miMic. The node shape represents its origin of significance: spheres were identified by both miMic and the Mann-Whiteny test on leaves, circles were identified by miMic only, and squares were identified by only the Mann-Whitney test. The colors represent the taxonomic family of each node.

Figure 6: miMic's plots - example on IBD cohort. A. Bar plot illustrating the taxonomy levels in the miMic test vs. the number of significant findings in a real run (RP) shown in blue, and in a shuffled run (SP) shown in red. The highest bar plot represents the actual RP vs. SP of the selected taxonomy level of miMic combined with the leaves test as explained in the Methods. Taxonomy levels used for the a priori nested ANOVA test are shaded in grey. The number of RP significantly exceeds the number of SP. B. Interaction between significant taxa found in miMic. Each taxon is colored according to its significant family color, similar to Fig. 5 above. Each node shape represents the taxon's order. An edge is drawn between two nodes if their SCC is above 0.3 (user- adjustable) and its p-value < 0.05. The width of the edge corresponds to its SCC. A blue edge represents a positive relation, while a red edge represents a negative one.

C. Analysis of significant positive and negative relations within taxonomic families. The y-axis displays significant families in the cohort (defined by a family that has at least 1 significant descendant), while the x-axis shows the count of positive relations within a family in blue or the count of negative relations within a family in red. Each family is colored according to its color in the interaction network in (B) and the cladogram of correlations in Fig. 5 above.

6) An open source implementation is provided with examples. I did not find the source code for the analyses and figures shown in this manuscript, however.

The miMic tool is readily accessible to users through various platforms, including a user-friendly PyPi package [6], a dedicated website [7], and a GitHub repository [8]. Additionally, we have ensured the availability of the complete source code used for the analyses and figures presented in the manuscript. You can find the source code in our GitHub repository https://github.com/oshritshtossel/miMic_all_analyses, see the GitHub readme for exact details. We encourage reviewers and users to explore the source code for a deeper understanding of our methods and results.

Minor:

- the text refers to "inner structure" and "inner relation" between microbial taxa; does this refer to covariance between microbial abundances, the phylogenetic relation, or both? I suggest to use the established terms for more clarity.

We have now removed the concept of inner structure from Table 1. Any relation between taxa is now explicated.

- "microbiota" refers to taxonomic composition and could be better justified for this manuscript than "microbiome" (see also Marchesi et al. 2015 for suggested vocabulary for microbiome research)

We have updated the term to 'microbiota' throughout the manuscript. Specifically, when treating the entire microbiome and its taxonomic composition, we use biota (for example in the title). When treating a set of microbes, we use the term microbiome. Paper mentions ANCOM and ANCOM-C (l. 243/53); does the latter refer to ANCOM- BC?

In our previous manuscript version, we only applied ANCOM for our analyses. ANCOM-C referred to ANCOM with multiple measurement corrections as explicitly explained in the revised manuscript. However, in response to the reviewer's suggestion, we have now incorporated ANCOM-BC2 into our revised manuscript, denoted as 'ANCOM-BC2' and 'ANCOM-BC2-C' when applied with multiple measurement correction. We apologize for any confusion regarding the terminology and have clarified this addition in the revised manuscript text. Thank you for bringing this to our attention; we appreciate your valuable feedback.

- apriori -> a priori

Thanks, we fixed it in the revised manuscript.

- Appendix, section 6 is empty?

We are sorry for this mistake the section on “Joint distribution” is an inner section of the Appendix. We fixed this mistake in the revised manuscript.

- Project website listed in the manuscript (https://micros.math.biu.ac.il/) does not open

Thanks, we fixed the link.

Reviewer 2:

The article introduces miMic, a novel approach for microbiome differential abundance analysis. Designed to tackle issues like sparse data, varying abundance scales, and taxonomic relationships, miMic operates through a structured process involving data preprocessing, apriori nested ANOVA, and a post hoc miMic test along cladogram trajectories. It aims to reduce false positives while maintaining high discovery rates. The validation includes analytical models, simulations, and comparisons with existing methods over diverse datasets, demonstrating miMic's accuracy and advantages. However, the definitive reason for considering the method a clear winner isn't evident to me. Here are my major comments:

1. The article underscores the difficulty of assessing differential analysis methods without a definitive ground truth, presenting a challenge in conclusively validating the proposed method's accuracy. It is noteworthy that the simulations are crafted to mirror the hierarchical structure of the proposed model, imparting a constructed advantage to the method in question. However, it is acknowledged that these simulations may not fully capture the characteristics of real microbiome data. To enhance the evaluation, the authors are encouraged to explore alternative simulations, such as utilizing the simulation function to mimic real data in the GUniFrac package in R.

We sincerely appreciate your thorough review of our manuscript and are grateful for your approval of our new statistical test, miMic. We have carefully considered and addressed the reviewer’s comments. First, we have added a section at the end of the Introduction that provides a concise explanation of miMic's underlying principles. The following text was added to the revised manuscript:” To introduce the relation between taxa in relative abundance analysis and simultaneously solve the multiple measurement corrections, we here introduce miMic (Mann-Whitney iMage Microbiome) - a novel framework to apply differential abundance analysis to the non-normally distributed microbial data.

Simply stated, on the one hand, applying a Bonferonni multiple measurement correction on all taxa is too stringent. On the other hand, applying a test on all taxa with no correction will produce a large number of false positive associations (basically the p-value multiplied by the number of taxa, which is often more than the number of biologically significant associations). However, formally, not all taxa are independent measurements, since the frequencies of similar taxa are often correlated. miMic uses these relations and a simplifying assumption that if a taxon is associated with a label, this effect is strong enough to affect the average of the taxa at coarser levels to reduce the number of required corrections. In practice, miMic performs the correction at coarse levels, where there are few taxa, and then only performs tests along significant paths in the cladogram.” (Lines 88-100).

The reviewer is right that the simulations are generic for hierarchical data and are not specific to microbial data. The rationale for using the hierarchical structure to reduce the false discoveries rate is not specific to the microbiome realm and can be applied to generic hierarchical data too. Therefore, the simple and generic simulation was chosen on purpose. However, as the reviewer mentions, realistic microbiome-oriented simulations are more appropriate to the current analysis, and we have added that by utilizing three distinct real microbial datasets, each varying in size (n=83, n=257, n=684). These datasets were chosen to fully capture the characteristics of real microbiome data. We randomly selected 10 taxa along with all their ASVs, and subsequently increased their abundances by 20% in samples exhibiting a phenotype of 1. This process was mirrored for another set of 10 taxa associated with a phenotype of 0. The assignment of phenotypes to samples was done randomly and uniformly. Then the F1 score of each model was computed (Fig. 2 M). One can clearly see in these simulations that miMic is much more precise than all current methods.

Figure 2: Validation of miMics' assumptions on analytical models and simulations. A. Daughter's distribution (pink) vs. mother's distribution (dark pink) in the regime of (0,0,0). The mother's distribution is noticeably narrower, with approximately half that of the daughter's distribution. B - D. Comparison of the leaf confidence with miMic's confidence based on analytical integral calculations over 3 different regimes: regime (0,0,0) (B), regime (0,0,μ) (C), regime (0,μ,αμ) (D). The lines represent estimated slopes, denoted as S. In D, different colors represent varying levels of connection between the sisters, controlled by α values (0.25, 0.5, 1, 2, 4). E. Histogram illustrating the distribution of inner sisters-label SCCs across different cohorts. The black line represents the zero line, and the dashed pink line represents the average of the distribution, indicating a right-skewed distribution, with most sisters showing a consistent positive correlation with the label. F - G. A comparison between the number of samples and the number of FP for miMic and Mann-Whitney leaf simulations based on the regime of (0, 0, 0) (F) and the regime of (0, 0, μ), where μ was set to 1. The lightest pink color represents the Leaf-C model, the middle pink represents the Leaf model, and the darkest pink represents the miMic model. H. Comparison between the number of samples and the number of TP for miMic and Leaf Mann-Whitney simulations based on the regime of (0, 0, μ). The color coding is similar to the color coding of F -G. I - J. Comparison between the FP (I) and TP (J) of the miMic model and the Leaf model in the simulation of the regime (0, 0, μ) over different numbers of samples and different values of μ.The Leaf model shows a higher number of FP compared to miMic, while miMic's TP taxa are similar to Leaf's TP taxa. K - L. Comparison between the FP (K) and TP taxa (L) of the miMic model and the Leaf model in the simulation of the regime (0, μ, αμ) over different numbers of samples and different values of α. The Leaf model exhibits a higher number of FP compared to miMic, while miMic's TP are similar to the Leaf's TP. M. The F1 scores of different differential abundance methods are depicted across three distinct setups of microbiome-oriented simulations. miMic is again pink and is much higher than all current methods, many of which have an F1 of 0.

2. While miMic is presented as versatile, the complexity of the method, involving multiple steps like data preprocessing, cladogram translation, apriori nested ANOVA, and post hoc miMic test, might be a drawback for users seeking simpler approaches. Specifically, the proposed method necessitates specific preprocessing steps, such as merging similar features based on taxonomy, scaling the distribution, standardizing to z-scores, and dimension reduction. Concerns are raised regarding the potential loss of candidates during the feature merging and dimension reduction stages. An important question arises: what would be the outcome if the preprocessing steps are omiXed, and miMic is applied directly to the data? Additionally, it would be valuable to know whether preprocessing was applied to the original data before subjecting it to other state-of-the-art methods.

While it is true that miMic involves several sequential procedures, each step is meticulously designed to contribute to miMic's performance in reducing false positives while preserving real positives. One fundamental aspect of miMic's methodology is its utilization of trajectories derived from the cladogram of means. As a result, raw ASVs cannot be directly employed, and they must be grouped into taxonomies. This grouping process can be achieved through various methods, such as summing, averaging, or applying Sub-PCA to the ASVs within each group. In our previous work [10], we have found that Sub-PCA merging often yields optimal results, followed by log normalization. Therefore, these preprocessing steps are recommended defaults within miMic. Note that the merging method and normalization do not drastically affect the results. Our experiments have demonstrated that the choice of merging method and normalization has a minor impact on results, as evidenced by slightly worse outcomes with the summing method and relative normalization (referred to as miMic relative) (see Fig. 3 B and C). Subsequently, the construction of cladograms is imperative, followed by the application of miMic's two-step testing approach. Initially, an a priori test leveraging the cladogram structure (nested ANOVA) is conducted, serving as a crucial filter to identify signals of interest. Only upon the detection of such signals is the hierarchical post-hoc miMic test applied.

Despite the multiple steps involved, miMic is designed to be user-friendly and accessible. We have developed a simple end-to-end PyPi package [7] and a dedicated website [9] that automates all necessary procedures, requiring only raw ASVs and phenotype data from the user. Thus, users can seamlessly apply miMic without the need for intricate manual intervention. There are detailed examples and explanations in the Supp. Mat about how to apply miMic.

Regarding the preprocessing of other state-of-the-art methods used for comparisons, we did not apply miMic's preprocessing steps. Instead, we followed the preprocessing steps outlined in the original papers for each respective method. This clarification has been added to the revised manuscript. In addition, we added a table that summarizes the different preprocessing required for each tool (Supp. Mat. Table S4).

Figure 3: Validation of miMic vs. SOTA models on real-world datasets. A. RSP(β) schematic explanation. The tasks in the differential analysis field can be divided into 2 main types: (1) tasks with a predefined GT, where False and True labels are clearly defined (upper left scheme), and (2) tasks without a predefined GT, as is common in most real-world datasets (upper right scheme). In the second scenario, "True" and "False" are replaced with "Real" (calculated on real labels) and "Shuffled" (calculated on shuffled labels). The RSP(β) score is defined by (β *RP -SP)/ (β *RP+SP)), where β representing the user-defined weighting of Real Positives (RP) vs. Shuffled Positives (SP). B - C. Comparative analysis of different differential analysis methods as a function of RSP(β) over 16S cohorts (B) and WGS cohorts (C). Each color represents a specific model: orange for DeSeq2 (light without FDR correction and dark with FDR correction, denoted as DeSeq2-C), yellow for LefSe, green for ANCOM (light without FDR correction and dark with FDR correction, referred to as ANCOM-C), blue for LINDA (light without FDR correction and dark with FDR correction, denoted as LINDA- C), brown for ada-ANCOM, and pink for miMic (pink for log Sub-PCA MIPMLP preprocessing and purple for relative mean MIPMLP preprocessing). Each line illustrates the average RSP(β) score across all cohorts (12 16S in B and 8 WGS in C) The light shadows surrounding each line represent standard errors calculated over 10 simulations of the shuffled models across all cohorts (12 16S in B and 8 WGS in C). D - E. Comparison of different starting taxonomy levels of the miMic test and their corresponding RSP(β) scores over 16S cohorts (D) and WGS cohorts (E). Each taxonomy level is indicated by a different line style (1 for kingdom, 2 for phylum, 3 for class, 4 for order, 5 for family, 6 for genus, and 7 for species). Typically, the best RSP scores are achieved in the first two taxonomy levels. The inner bar plot presents the number of cohorts in which miMic is deemed significant when commencing the Mann-Whitney test at each taxonomy level. F. Scatter plot of the sister's-labels SCC vs. the RSP(1) score. A significant positive correlation of 0.588 is observed between the SCCs of sister labels and the model's performance

Table S4: Different preprocessing of different tools

3. The validation of miMic is based on analytical models, simulations, and real-world cases. However, the article should discuss potential limitations or cases where miMic may not perform optimally. The method's boundaries and capabilities have not been thoroughly elucidated.

An important limitation of miMic is the identification of rare taxa that are highly significant but have minimal contribution to the upper taxonomy levels. To address this limitation, we have developed an enhanced version of miMic. In this new iteration, we first apply the original miMic to identify consistently significant taxa within their taxonomic families. Subsequently, we employ the Mann-Whitney test with a multiple measurement correction exclusively on the leaves to identify from the rare taxa those that are exceptionally significant. Finally, we report the union of significant taxa identified by both methods. To provide concrete examples and illustrate the performance differences between the original and enhanced versions of miMic, we have conducted comprehensive comparisons. In the IBD cohort, for instance, both versions yield an RSP of 1, as measured by the previous miMic and the enhanced version. However, discernible differences emerge when examining Fig. 5 vs. Fig. R1, where taxa recognized by miMic and leaves are represented by spheres, taxa recognized by leaves alone are depicted as squares, and taxa recognized by the original miMic are indicated by circles. Furthermore, we have included the previous tree visualization of the IBD cohort in the revised manuscript, highlighting specific differences. For instance, the Coprococcus catus species among others, identified as significant by the revised miMic, was not deemed significant by the previous version. Notably, the identification of additional significant taxa does not increase the count of shuffled positives, which remains at 0. We have also enhanced the visualization from the previous version, showcasing only significant leaves and their descendants, and introducing a colorful mode that classifies taxa based on their family color.

Another limitation of miMic is that when finer taxonomy levels exhibit opposite relationships with the phenotype, the contributions of these levels may cancel each other out at higher taxonomy levels where values are averaged.

However, the main limitations of miMic emerge from the inherent analysis of microbiome samples that have a very limited overlap between datasets. Indeed, the fraction of taxa overlapping between any two datasets is typically less than 0.1 [6]. Thus, differential expression analysis in per-definition is often not well transferred between datasets. However, the requirement of consistent associations along the cladogram trajectory ensures that at least at a coarse taxonomical description, such association can be maintained. To explain that, we have expanded the limitations section in the revised paper to provide a more comprehensive overview of miMic's boundaries and capabilities. The added text reads as follows: “Since miMic tests either observed taxa or taxa consistently significant along the cladogram, the main associations missed by it are rare microbe associations at coarse taxonomy levels. For example, assume two sister genera with precise associations with a label. Their mother taxon will not be detected by miMic. However, such cases are rare, since 1) Most sisters' frequencies are positively and not negatively correlated. 2) Even in the case of opposite associations, two opposing associations will cancel one another only if they are of the same order. Indeed, in the example presented here, we show the detection of such opposite associations. Moreover, to address that we propose to start miMic at different levels. However, consistently the phylum level or the kingdom level gave the highest RSP scores. However, the main limitations of miMic emerge from the inherent analysis of microbiome samples that have a very limited overlap between datasets. Indeed, the fraction of taxa overlapping between any two datasets is typically less than 0.1 [6]. Thus, differential expression analysis in per-definition is often not well transferred between datasets. However, the requirement of consistent associations along the cladogram trajectory ensures that at least at a coarse taxonomical description, such association can be maintained.” (Lines 377-391)

Figure 5: Differential abundance analysis results are visualized on a cladogram, where each color represents the sign of the Mann-Whitney score (blue for positive scores, red for negative scores, and grey for non-significant taxa). The node size corresponds to -log10(p-value) from the Mann-Whitney test in miMic. The node shape represents its origin of significance: spheres were identified by both miMic and the Mann-Whiteny test on leaves, circles were identified by miMic only, and squares were identified by only the Mann-Whitney test. The colors represent the taxonomic family of each node.

Figure R1: Differential abundance analysis results are visualized on a cladogram, where each color represents the sign of the Mann-Whitney score (blue for positive scores, red for negative scores, and grey for non-significant taxa). The node size corresponds to -log10(p-value) from the Mann-Whitney test in original miMic.

4. The article notes that miMic obtains the best results when starting on specific taxonomy levels. This sensitivity to the cladogram structure may limit its generalizability to diverse datasets.

We appreciate the reviewer's concern. However, our findings indicate that miMic achieves optimal results when it begins testing at coarser taxonomy levels, such as the kingdom or phylum (see above Fig. 3 D and E). By initiating the test at the coarsest taxonomy level and subsequently narrowing it down, miMic demonstrates a high level of generality across diverse datasets. This approach ensures that miMic is not overly specific but rather adaptable to varying taxonomic structures present in different datasets.

We have taken additional steps to assess miMic's generality and robustness across various dataset characteristics. We conducted a thorough examination of miMic's performance in relation to dataset attributes such as read depth (variance and median), sparsity, richness, percentage below prevalence cutoff, and sample size. This analysis involved computing the Spearman correlation between these dataset characteristics and both the percentage of significant features and the RSP(1) score (Supp. Mat. Fig. S5). These comprehensive evaluations underscore miMic's versatility and reliability across a wide range of dataset compositions and characteristics (see also our answer to Reviewer 1 (4)).

In summary, miMic's approach of initiating tests at coarser taxonomy levels enhances its generalizability to diverse datasets, while our extensive evaluations confirm its robustness and adaptability to different dataset characteristics. We hope this clarification addresses the reviewer's concerns and reinforces miMic's suitability for a broad spectrum of microbiome analysis applications.

Figure S5: Sensitivity robustness assessment. A-B. The heatmaps illustrate Spearman correlation coefficients (SCCs) between each generic dataset characteristic and the percentage of significant taxa identified by each tool per dataset in 16S (A) and WGS (B) cohorts. C-D. The heatmaps illustrate Spearman correlation coefficients (SCCs) between each generic dataset characteristic and the RSP(1) score per dataset in 16S (C) and WGS (D) cohorts. Positive correlations are depicted in red, while negative correlations are shown in blue. Stars indicate a significant correlation (p-value < 0.05). miMic demonstrates robustness across all tested generic features in 16S and WGS datasets.

5. While the article emphasizes the importance of considering the correlation structure among taxa, it would be useful to discuss how miMic handles cases where taxa have complex and nonlinear relationships.

We have reported two main limitations of miMic. First, when lower taxonomy levels exhibit opposite relationships with the phenotype, the contributions of these levels may cancel each other out at higher taxonomy levels where values are averaged. Second, extremely rare taxa whose contributions to higher levels are minor may not be adequately reflected. To address the latter limitation, we have expanded miMic to include significant leaves identified through the Mann-Whitney test, with multiple measurements correction using Bonferroni adjustment. By doing so, we ensure that only rare taxa in the leaves are not omitted from the analysis. This still leaves the case of the intermediate level vertices (e.g. genera where the species are not significant, and the family are not significant, but their genera is). Such cases are indeed missed by miMic, and this is explained and clarified.

6. The challenge of differential analysis varies between the majority and minority microbiome. It appears that the proposed method excels in detecting abundant features compared to scarce features. Because the paper mentioned it exhibits more power in identifying upper-level nodes than lower-level nodes. Is this true? Are there any validations?

This is indeed correct, features that do not affect higher levels, since they are too rare may be missed. We have now partially fixed that by adding the test on the leaves. While this is indeed a limitation of miMic, the gain in reducing false positives is more important than the few cases of association with rare microbes that are not the leaves of the taxonomic tree.

7. The theoretical calculations are somewhat perplexing. In Equation (3) of section 7.1, m- significant is denoted, while in Equation (10) of section 7.2, f-significant is mentioned. It's unclear what f represents in this context. Moreover, the assertion that from the joint distribution, one can conclude with high probability that if sisters are not associated with the class, neither is their mother, seems non-trivial. Please elaborate it in more details.

We sincerely apologize for the typo in our manuscript regarding the term 'f-significant.' The correct term should have been 'm-significant,' and we appreciate the reviewer for bringing this to our attention. We have rectified this error in the revised version of the manuscript to ensure accuracy and clarity. Regarding the non-triviality of the assertion that from the joint distribution, one can conclude with high probability that if sisters are not associated with the class, neither is their mother. We clarified in the revised manuscript analytically that if the sisters are not associated with the class ~N(0,1) then their mother distribution is ~N(0, 1 √3), which has a lower variance than the daughters’ distributions. Therefore, the probability that it will be observed as significant is even lower than the one of the daughter. Moreover, numerical results show that requiring a p-level significance on both the mother and the daughter is approximately equivalent to a 𝑝2 requirement on the daughter. As such, the fraction of SP cases is much lower than p (see above Fig. 2 B). We hope the assertion is now clearer. The revised text that was added to the revised manuscript: “In this simplified model, one can analytically show (See appendix and Fig. 2 A) that if the sisters are not associated with the class ~N(0,1), then their mother distribution is ~N(0, 1 √3). As such, the probability that it will be observed as significant is even lower than the one of the daughter (formally, this is equivalent to having 6S-2 degrees of freedom (DOF) and doing a test with 2S-2 DOF, where S is the number of samples). Moreover, numerical results show that requiring a p level significance on both the mother and the daughter is approximately equivalent to a 𝑝2 requirement on the daughter. As such, the fraction of SP cases is much lower than p (see Fig. 2 B).” (Lines 212-219).

8. In the captions of Figure 4, plot E is not described. The current descriptions for plot E are meant for plot F. Please check the captions and descriptions of all the other figurees

We apologize for the oversight regarding the figures and their captions in the initial submission. We have rectified this error in the revised manuscript by ensuring that both the figures and their captions are accurate and appropriately labeled. We redesigned Fig. 4 in the revised manuscript. It has been separated into two different figures in the revised manuscript, Fig. 5 (see above) and Fig. 6 (see below). Additionally, two of its subplots were moved into the Supplementary Material for clarity. Thank you for bringing this to our attention, and we appreciate your diligence in reviewing our work

Figure 6: miMic's plots - example on IBD cohort. A. Bar plot illustrating the taxonomy levels in the miMic test vs. the number of significant findings in a real run (RP) shown in blue, and in a shuffled run (SP) shown in red. The highest bar plot represents the actual RP vs. SP of the selected taxonomy level of miMic combined with the leaves test as explained in the Methods. Taxonomy levels used for the a priori nested ANOVA test are shaded in grey. The number of RP significantly exceeds the number of SP. B. Interaction between significant taxa found in miMic. Each taxon is colored according to its significant family color, similar to Fig. 5 above. Each node shape represents the taxon's order. An edge is drawn between two nodes if their SCC is above 0.3 (user-adjustable) and its p-value < 0.05. The width of the edge corresponds to its SCC. A blue edge represents a positive relation, while a red edge represents a negative one. C. Analysis of significant positive and negative relations within taxonomic families. The y-axis displays significant families in the cohort (defined by a family that has at least 1 significant descendant), while the x-axis shows the count of positive relations within a family in blue or the count of negative relations within a family in red. Each family is colored according to its color in the interaction network in (B) and the cladogram of correlations in Fig. 5 above.

Reference:

1. Nearing, Jacob T., et al. "Microbiome differential abundance methods produce different results across 38 datasets." Nature Communications 13.1 (2022): 342.

2. Sankaran, Kris, and Susan Holmes. "structSSI: simultaneous and selective inference for grouped or hierarchically structured data." Journal of statistical software 59.13 (2014): 1. 3. https://github.com/mruehlemann/phaat

4. Zhou, Chao, Hongyu Zhao, and Tao Wang. "Transformation and differential abundance analysis of microbiome data incorporating phylogeny." Bioinformatics 37.24 (2021): 4652- 4660.

5. Weiss, Sophie, et al. "Normalization and microbial differential abundance strategies depend upon data characteristics." Microbiome 5 (2017): 1-18.

6. Shtossel, Oshrit, et al. "Gut microbiome-metabolome interactions predict host condition." Microbiome 12.1 (2024): 1-19.

7. https://pypi.org/project/mimic-da/

8. https://github.com/oshritshtossel/miMic

9. https://micros.math.biu.ac.il

10. Jasner, Yoel, et al. "Microbiome preprocessing machine learning pipeline." Frontiers in Immunology 12 (2021): 677870.

11. Duvallet, Claire, et al. "Meta-analysis of gut microbiome studies identifies disease-specific and shared responses." Nature communications 8.1 (2017): 1784.

12. Xia, Y., et al. "Combined analysis of metagenomic data revealed consistent changes of gut microbiome structure and function in inflammatory bowel disease." Journal of Applied Microbiology 131.6 (2021): 3018-3031
